# Hypoxia-inducible factors: cancer progression and clinical translation

**DOI:** 10.1172/JCI159839

**Published:** 2022-06-01

**Authors:** Elizabeth E. Wicks, Gregg L. Semenza

**Affiliations:** 1 Department of Genetic Medicine,; 2 Institute for Cell Engineering, and; 3 Stanley Kimmel Comprehensive Cancer Center, Johns Hopkins University School of Medicine, Baltimore, Maryland, USA.

## Abstract

Hypoxia-inducible factors (HIFs) are master regulators of oxygen homeostasis that match O_2_ supply and demand for each of the 50 trillion cells in the adult human body. Cancer cells co-opt this homeostatic system to drive cancer progression. HIFs activate the transcription of thousands of genes that mediate angiogenesis, cancer stem cell specification, cell motility, epithelial-mesenchymal transition, extracellular matrix remodeling, glucose and lipid metabolism, immune evasion, invasion, and metastasis. In this Review, the mechanisms and consequences of HIF activation in cancer cells are presented. The current status and future prospects of small-molecule HIF inhibitors for use as cancer therapeutics are discussed.

## Hypoxia-inducible factors and oxygen homeostasis

Oxygen homeostasis represents one of the most daunting and most essential challenges facing humans: to precisely supply, on a continuous basis, adequate O_2_ to each of the approximately 50 trillion cells in the adult body to meet their metabolic demands for oxidative phosphorylation and several hundred other biochemical reactions that require O_2_ (1). Adding to the complexity of this challenge, cells throughout the body reside in tissue microenvironments with dramatically different O_2_ levels: airway epithelial cells are exposed to 21% O_2_, whereas in mouse thymus the median recorded partial pressure of oxygen (pO_2_) was 7.6 mmHg, which corresponds to approximately 1% O_2_ (2). Even within the same organ, tissue oxygenation varies tremendously: in the kidney, pO_2_ varies from 70 mmHg in the outer cortex to 10 mmHg in the inner medulla (3).

At the transcriptional level, the challenge to maintain oxygen homeostasis is met by the action of hypoxia-inducible factors (HIFs), which mediate reprogramming of each cell’s transcriptome in response to decreased O_2_ availability (i.e., hypoxia). HIFs modulate the balance between oxidative and glycolytic metabolism as a means of matching O_2_ demand with available supply (4, 5) and stimulate increased O_2_ delivery by activating the transcription of genes controlling erythropoiesis (6, 7) and angiogenesis (8, 9) to increase systemic and local O_2_ supply, respectively. Within any given cell subjected to hypoxia, the expression of hundreds to thousands of genes will be increased or decreased. For example, when SUM159 human breast cancer cells were transferred from a standard tissue culture incubator containing 95% air and 5% CO_2_ (i.e., 20% O_2_) to a chamber containing 1% O_2_ for 24 hours, expression levels of 1307 RNAs were significantly increased and of 817 RNAs were significantly decreased more than 1.5-fold in a HIF-dependent manner, i.e., these changes were not observed in cells in which HIF expression was silenced (10). Hypoxia-induced RNA expression is due to direct binding of HIFs to hypoxia response elements (HREs) in target genes, which contain the core HIF-binding site sequence 5′-(A/G)CGTG-3′ (11). In contrast, hypoxia-repressed RNA expression is indirectly mediated by the HIF-dependent activation of genes encoding microRNAs, transcriptional repressors, chromatin-modifying proteins, and proteins that modify or bind to RNA (12–15).

HIFs are heterodimeric proteins consisting of an O_2_-sensitive HIF-1α, HIF-2α, or HIF-3α subunit and a constitutively expressed HIF-1β subunit (also known as ARNT) (16). The mechanism by which changes in O_2_ availability are transduced to HIF-mediated changes in gene expression is remarkably straightforward: under normoxic conditions, an oxygen atom is inserted into a proline residue of HIF-1α, HIF-2α, or HIF-3α by one of three HIF prolyl hydroxylases (PHD1, PHD2, PHD3), and the von Hippel-Lindau (VHL) protein binds selectively to hydroxylated HIF-α subunits, targeting them for ubiquitination and proteasomal degradation, whereas under hypoxic conditions, hydroxylation is inhibited and non-hydroxylated HIF-α subunits accumulate, dimerize with HIF-1β, and bind to HREs in target genes to activate transcription (16). HIF transcriptional activity is further modulated by factor inhibiting HIF-1 (FIH-1), which hydroxylates an asparagine residue in the transactivation domain of HIF-α subunits, thereby blocking binding of the coactivator proteins p300 and CBP (17, 18). Thus, prolyl and asparaginyl hydroxylation of HIF-α subunits negatively regulates their half-life and transcriptional activity, respectively, in an O_2_-dependent manner.

The critical role of the HIF pathway in maintaining oxygen homeostasis is illustrated by the genetic condition known as familial erythrocytosis, in which affected individuals have increased red blood cell production. Affected individuals carry germline mutations in the gene encoding the erythropoietin receptor (*EPOR*), erythropoietin (*EPO*), HIF-2α (*EPAS1*), PHD2 (*EGLN1*), or VHL (6). Whereas mutations in the *EPO* or *EPOR* gene result in erythrocytosis only, mutations in *EPAS1*, *EGLN1*, or *VHL* increase HIF-1 and/or HIF-2 activity in every cell of the body and result in additional phenotypic manifestations, including pulmonary hypertension (19) and predisposition to thromboembolic events (20).

Familial erythrocytosis is a rare genetic disorder, whereas ischemic cardiovascular disease is one of the most common causes of mortality in the United States. The age-related impairment of vascular remodeling in response to ischemia that plays a critical role in the pathogenesis of this disorder is due in part to an age-related impairment of HIF activation (21). By contrast, increased HIF activity contributes to the pathogenesis of cancer, another major cause of mortality, as will be discussed in detail below.

## Mechanisms of HIF activation in cancer

Many advanced human cancers contain regions of intratumoral hypoxia: the median pO_2_ in cancers of the breast, cervix, and head/neck is 10 mmHg (~1.4% O_2_), with one-quarter of all measurements falling between 0 and 2.5 mmHg (22). Indeed, even preinvasive lesions, such as ductal carcinoma in situ of the breast, may contain regions of necrosis (23) in which O_2_ availability is insufficient to maintain cell viability (24). In human breast cancers, measured diffusion distances for O_2_ ranged from 70 μm at arterial inflow to 30 μm at venous outflow (3), meaning that O_2_ rapidly becomes limiting as distance from the nearest blood vessel increases. Patients with cervical cancer, head/neck cancer, or soft-tissue sarcoma who have intratumoral pO_2_ less than 10 mmHg have significantly decreased survival (22). Intratumoral hypoxia is a stimulus for the induction of HIF-1α and HIF-2α protein expression, and increased expression of one or both of these proteins, as detected by immunohistochemical analysis of the diagnostic tumor biopsy, is associated with increased patient mortality in a wide range of solid cancers and leukemias (*n =* 101 studies; Supplemental Table 1; supplemental material available online with this article; https://doi.org/10.1172/JCI159839DS1). Whereas some cancers show a pattern of HIF-1α expression that is hypoxia-induced, in which cells furthest away from a blood vessel show the highest expression, in other cancers a homogeneous increase in HIF-1α expression is detected by immunohistochemistry, suggesting that an O_2_-independent mechanism is responsible for increased expression (25).

The most dramatic example of O_2_-independent HIF-1α and HIF-2α protein expression occurs in tumors associated with the von Hippel-Lindau syndrome, in which affected individuals are heterozygous for a germline loss-of-function mutation in the *VHL* tumor suppressor gene and the other allele is inactivated in the tumor tissue, leading to development of the clear-cell type of renal cell carcinoma (RCC), central nervous system and retinal hemangioblastoma, pancreatic neuroendocrine tumor, and other neoplasms (26). In these VHL-null tumors, cancer cells are strongly positive for HIF-1α and/or HIF-2α by immunohistochemistry (27, 28). In contrast to the partial loss-of-function mutations in *VHL* that cause erythrocytosis in the homozygous state but retain sufficient HIF binding activity to suppress tumor formation, heterozygosity for a *VHL* loss-of-function mutation is not sufficient to cause erythrocytosis, but loss of the second allele in the tumor results in VHL activity that is insufficient to suppress tumor formation. Loss of function for other tumor suppressors that frequently occurs in tumors due to somatic mutation or methylation, including p53 (29) and PTEN (30, 31), has also been reported to increase HIF-1α expression in one or more cancer types. Activation of receptor tyrosine kinases, such as the epidermal growth factor receptor (EGFR) and human epidermal growth factor receptor 2 (HER2), leads to increased mTOR activity and increased HIF-1α mRNA translation into protein in prostate (30) and breast (32) cancer, respectively. Many noncoding RNAs have been shown to dysregulate HIF-1α expression in cancer cells (33). Thus, genetic alterations and intratumoral hypoxia contribute in varying degree to the high levels of HIF-1α or HIF-2α that are observed in many human cancers.

## Consequences of HIF activation in cancer

Whereas any given cancer cell will express only a subset of the large battery of HIF-regulated RNAs (>7000 identified to date), in aggregate these RNAs contribute to every critical aspect of cancer progression, including tumor vascularization, metabolic reprogramming, cell motility and invasion, and resistance to chemotherapy and radiation therapy (34–36). Most recently, HIFs have been shown to play major roles in cancer stem cell specification and immune evasion (33, 37, 38). Increased HIF activity in both cancer and stromal cells plays a critical role in immune evasion (39–42). The descriptions below are representative rather than comprehensive accounts of the thousands of HIF target genes expressed in human cancers. Metastasis is not listed as a separate category because it is dependent on all of the processes described below (43–45).

### Vascularization.

HIFs activate the expression of multiple angiogenic growth factors that contribute to intratumoral blood vessels, including vascular endothelial growth factor (VEGF), stromal-derived factor 1 (SDF1; also known as CXCL12), stem cell factor (also known as KIT ligand), placental growth factor, angiopoietin 2, angiopoietin-like 4, and other secreted factors that stimulate angiogenesis locally as well as serving to recruit bone marrow–derived angiogenic cells that participate in tumor vascularization (8, 21, 35, 46, 47). The expression of this large battery of genes provides a molecular basis for the frequent failure of anti-VEGF therapy to effectively block tumor angiogenesis and growth. Furthermore, to the extent that anti-VEGF therapy is successful in inhibiting angiogenesis, it increases intratumoral hypoxia, which may stimulate increased invasion and metastasis (48, 49) by mechanisms that will be described below. These observations suggest that safe and effective use of angiogenesis inhibitors may require coadministration of a HIF inhibitor.

### Metabolic reprogramming.

**Figure 1:**
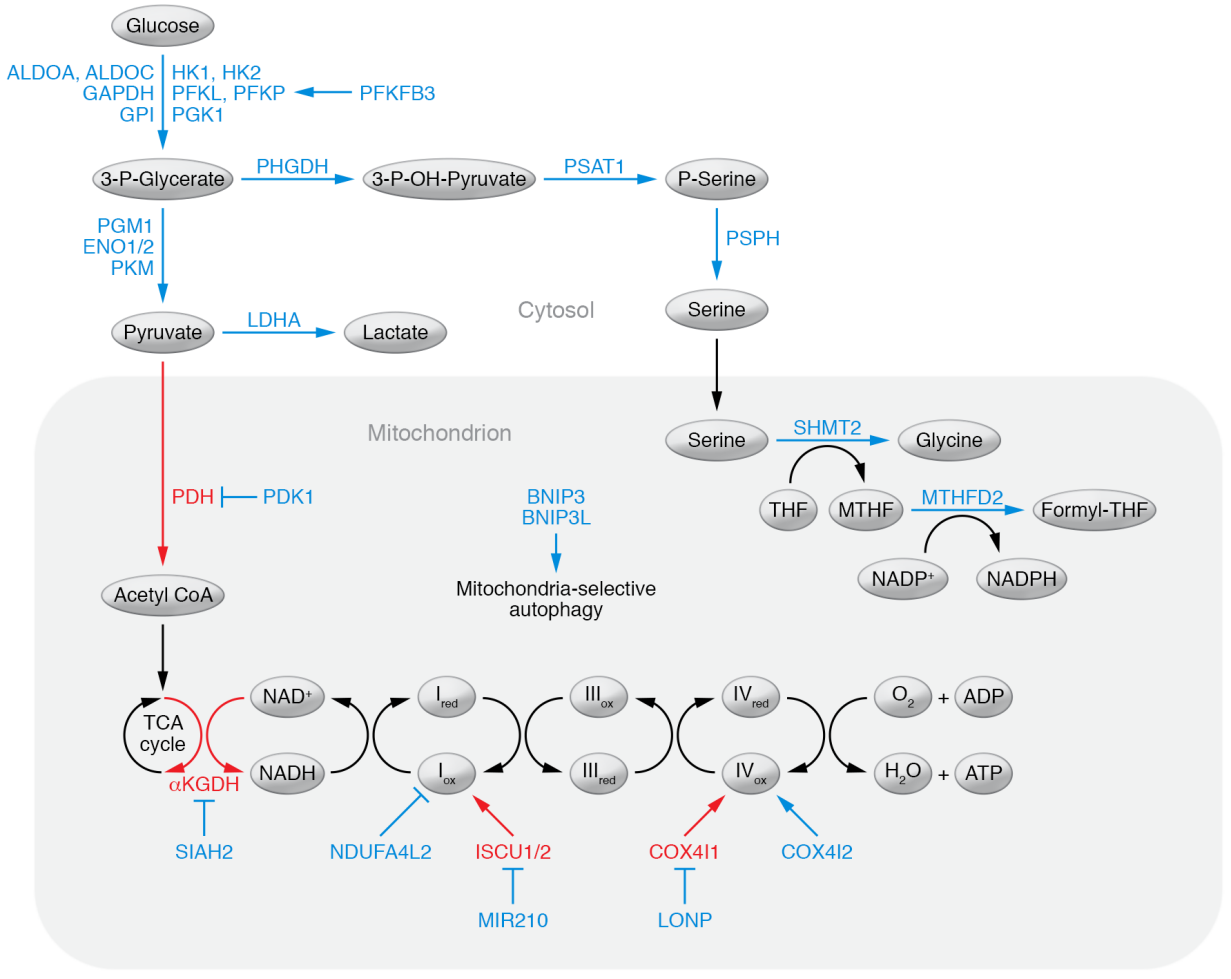
HIF target genes that regulate glucose metabolism. HIF target genes that are induced under hypoxic conditions, leading to increased glycolysis and/or decreased oxidative phosphorylation, are shown in blue. Genes that promote oxidative metabolism are shown in red.

HIF-1 plays a critical role as master regulator of the balance between oxidative and glycolytic metabolism (50). It does so by activating the expression of over two dozen genes (Figure 1). Perhaps foremost among these are *PDK1* (51, 52), encoding pyruvate dehydrogenase (PDH) kinase, which phosphorylates and inactivates the catalytic subunit of PDH, the enzyme that converts pyruvate to acetyl-CoA for entry into the tricarboxylic acid (TCA) cycle; and *LDHA* (11, 53), encoding lactate dehydrogenase, which converts pyruvate to lactate. Increased expression of PDK1 and LDHA shifts the balance of glucose metabolism to augment glycolysis and decrease mitochondrial respiration (51–53). Coordinate regulation of the genes encoding glucose transporters and glycolytic enzymes by HIF-1 (54) increases glucose flux through glycolysis as partial compensation for the reduced ATP yield compared with oxidative phosphorylation. Glycolytic flux is also stimulated by HIF-dependent expression of 6-phosphofructo-2-kinase/fructose-2,6-bisphosphatase-3 (PFKFB3), an enzyme that converts fructose-6-phosphate to fructose-1,6-bisphosphate, which is an allosteric inducer of phosphofructokinase (55).

Conventional wisdom held that cells switch from oxidative to glycolytic metabolism under hypoxic conditions in order to maintain ATP production. However, analysis of mouse embryo fibroblasts (MEFs) with knockout (KO) of HIF-1α led to a paradigm shift in our understanding of this metabolic adaptation. Unlike wild-type cells, HIF-1α–KO MEFs did not survive when cultured at 1% O_2_ for 3 to 4 days (51, 56). The HIF-1α–KO MEFs did not die as a result of ATP depletion: remarkably, ATP levels were higher in KO cells exposed to 1% O_2_ than in wild-type cells maintained at 20% O_2_. Clearly, the prevailing view that cells switched their metabolism because O_2_ was limiting for oxidative phosphorylation at 1% O_2_ was not correct. Instead, HIF-1α–KO MEFs died under long-term hypoxic conditions as a result of overwhelming levels of reactive oxygen species (ROS) (51, 56). HIF-1α–KO MEFs lost the ability to increase PDK1 expression in response to hypoxia, and their survival was rescued by forced expression of PDK1, which ameliorated ROS production under hypoxic conditions (51).

Life with O_2_ is a double-edged sword: When used as the terminal electron acceptor in respiration, it provides a mechanism for highly efficient ATP production. However, this process must be precisely modulated, because if the flow of electrons into the electron transport chain (ETC) at complex I is greater than the conversion of O_2_ to H_2_O at complex IV, electrons will spill out of complex I or III and combine with O_2_ non-catalytically to form superoxide anion. Under such circumstances, there are three possible adaptive responses: (a) decrease the flow of reducing equivalents from the TCA cycle to the ETC (e.g., by increasing PDK1 expression); (b) increase the activity of cytochrome *c* oxidase (complex IV); or (c) increase mitochondrial antioxidant capabilities to counteract the increased ROS production. In fact, all three of these strategies are employed by human cancer cells.

The most draconian measure employed to limit mitochondrial ROS production under hypoxic conditions is to eliminate mitochondria altogether, a process known as mitochondria-selective autophagy, thereby reducing oxidative metabolism of both glucose and fatty acids, which is mediated by HIF-dependent expression of the mitochondrial proteins BNIP3 (56) and BNIP3L (57). As described above for PDK1, the induction of BNIP3 expression in response to hypoxia is lost in HIF-1α–KO MEFs, and the cells can be rescued from ROS toxicity by forced expression of BNIP3 (56). Other HIF target genes that are expressed in order to maintain redox homeostasis include alternative subunits of ETC complex I (NDUFA4L2) and complex IV (COX4I2) that serve to either decrease the flow of electrons into the ETC at complex I (58) or increase the efficiency of electron transfer out of the ETC to O_2_ at complex IV (59), respectively. HIF-1–mediated expression of the microRNA miR-210 inhibits the expression of the iron-sulfur cluster scaffold proteins ISCU1 and ISCU2, which are required for complex I assembly, thereby decreasing mitochondrial respiration (60, 61). HIF-1 can also block the production of reducing equivalents in the TCA cycle by increased expression of SIAH2, which ubiquitinates the TCA cycle enzyme α-ketoglutarate dehydrogenase, targeting it for degradation (62). In breast cancer, the serine synthesis pathway and mitochondrial one-carbon metabolism are coordinately induced under hypoxic conditions to increase the generation of mitochondrial NADPH, which is required to maintain levels of reduced glutathione that are essential for counteracting the effects of increased ROS production (63). HIF-1 also controls glycogen synthesis and glycogenolysis, another metabolic cycle that plays a critical role in preventing toxic ROS production by cancer cells (64). Thus, HIFs function as master regulators for maintenance of ROS homeostasis in cancer cells under hypoxic conditions (Figure 1).

### Epithelial-mesenchymal transition.

A critical step in cancer progression is the epithelial-mesenchymal transition (EMT), in which cells lose the immobile epithelial phenotype characterized by a rigid cytoskeleton and extensive cell-cell interactions and take on a mesenchymal phenotype characterized by motility, which is enabled by a fluid cytoskeleton, loss of cell-cell interactions, and increased interactions with, and remodeling of, the extracellular matrix (ECM). Many HIF target genes contribute to each of these changes (43). In many cancers, EMT is controlled by a group of transcriptional repressors, including SNAIL, SLUG, TWIST, ZEB1, and ZEB2 (65), which downregulate the expression of E-cadherin and other epithelial cell–specific genes, and one or more of these repressors are HIF-regulated in many cancers (66–80). The HIF-dependent expression of many signaling proteins also promotes EMT (Supplemental Table 2).

### Cell motility.

Cell motility is triggered by members of the Rho family of GTPases, which mediate polymerization of actin stress fibers and serve as allosteric regulators of Rho-associated coiled-coil–forming kinase (ROCK) activity: GTP-loaded Rho binds (a) to ROCK and myosin phosphatase to stimulate phosphorylation and inhibit dephosphorylation of myosin light chain, respectively, leading to actin-myosin contraction; and (b) to LIM kinase to inhibit actin depolymerization (81–84). Hypoxia increases the motility of breast cancer cells, and this response is lost when expression of HIF-1α and HIF-2α is silenced (85). HIFs coordinately activate *RHOA* and *ROCK1* expression to stimulate the motility of hypoxic breast cancer cells (85). Cell motility requires the transmission of force through focal adhesions, which are correlated with cell velocity and regulated by focal adhesion kinase (FAK). Exposure of breast cancer cells to hypoxia induces FAK phosphorylation/activation and focal adhesion formation in a HIF-dependent manner (85). HIF-1 and HIF-2 also activate transcription of the *ADAM12* gene, which encodes a protease that specifically clips the extracellular domain of heparin-bound EGF-like growth factor (HB-EGF), which binds to EGFR, leading to FAK phosphorylation; and ADAM12 knockdown in breast cancer cells is sufficient to block hypoxia-induced random motility, directed migration, and ECM invasion in vitro and metastasis from breast to lungs in vivo (86).

### ECM remodeling.

Cancer cells degrade and modify existing ECM, altering its biochemical and biophysical properties to facilitate tumor growth, tissue invasion, and distant metastasis (87–89). Cancer cells lay down highly cross-linked collagen fibers that serve as a stiff track for rapid cell migration, and HIFs mediate the expression of a wide range of collagens, including the fibrillar and fibrillar-like collagens I, V, XI, and XXVII; network-forming/basement membrane collagens IV, VII, X, XV, and XVIII; filament-forming collagen VI; fibril-associated collagens IX, XIV, and XVI; transmembrane collagen XIII; and unclassified collagen XXVIII (10, 90–95); as well as collagen-modifying enzymes, including the lysyl oxidases LOX, LOXL2, and LOXL4; procollagen prolyl 4-hydroxylases P4HA1 and P4HA2; and procollagen-lysine, 2-oxoglutarate dioxygenases PLOD1 and PLOD2 (10, 93–103). In an orthotopic mouse model of breast cancer, metastasis from the mammary fat pad to the lungs was eliminated by knockdown of expression of P4HA1 or P4HA2 (97). Local tumor invasion and lung metastasis were also impaired by PLOD2 knockdown in breast cancer cells (99). The final stage of collagen fiber formation occurs following secretion of collagen fibrils into the extracellular space and is mediated by lysyl oxidases. These enzymes are not only secreted out of the cell but access the vasculature and travel to the lungs, liver, and other sites of metastasis, where they cross-link collagen and attract CD45^+^CD11b^+^ and VEGFR1^+^ myeloid cells to establish a pre-metastatic niche (103–105). In an orthotopic mouse model, cross-linked collagen and CD45^+^CD11b^+^ cells were detected in the lungs on days 7 and 14 after breast cancer cell implantation in the mammary fat pad, whereas cancer cells were not detected in the lungs until day 21 (103). Treatment of tumor-bearing mice with acriflavine or digoxin, drugs that inhibit HIF activity (47, 106), blocked collagen cross-linking, myeloid cell recruitment, and lung metastasis (107).

Cancer cells interact with ECM components through increased expression of integrins on their cell surface, and a remarkable number of genes encoding α-integrins (*ITGA1*, *ITGA2*, *ITGA5*, *ITGA6*, *ITGAV*, *ITGAX*) and β-integrins (*ITGB1*, *ITGB2*, *ITGB4*, *ITGB5*), which interact with collagen, fibronectin, and other ECM proteins, are induced by hypoxia in a HIF-dependent manner (90, 92, 93, 108–114). Knockdown of ITGA5 expression in breast cancer cells decreased their three-dimensional migration in collagen and within a multicellular spheroid, and inhibited lung metastasis without affecting primary tumor growth, in both human xenograft and mouse syngeneic orthotopic transplantation models (109). In addition to accessing blood vessels for metastasis to distant organs, breast cancer cells also invade lymphatic vessels and colonize lymph nodes. Many of the HIF-regulated genes described above that affect cell motility, tissue invasion, and lung metastasis also affect metastasis of breast cancer cells to the regional lymph node. HIFs also promote the formation of breast cancer lymphatic vessels through expression of platelet-derived growth factor B (115).

### Cancer stem cell specification.

Cancer stem cells (CSCs) are defined by their self-renewal and tumor-initiating properties (116, 117). CSCs are specified by their expression of a group of transcription factors that were initially identified in embryonic stem cells as pluripotency factors: Krüppel-like factor 4 (KLF4), octamer-binding transcription factor 4 (OCT4), SRY-box 2 (SOX2), and NANOG (118–121). Although CSCs cannot give rise to every cell type found in the body (the definition of pluripotency), upon mitosis they do give rise to two different cell types: one transit-amplifying cancer cell, which can divide rapidly but only for a limited number of mitoses, and one CSC, such that the number of CSCs is never diminished. Because of the limited proliferative capacity of the bulk tumor cells, it is believed that only CSCs give rise to clinically relevant recurrent and/or metastatic tumors.

**Figure 2:**
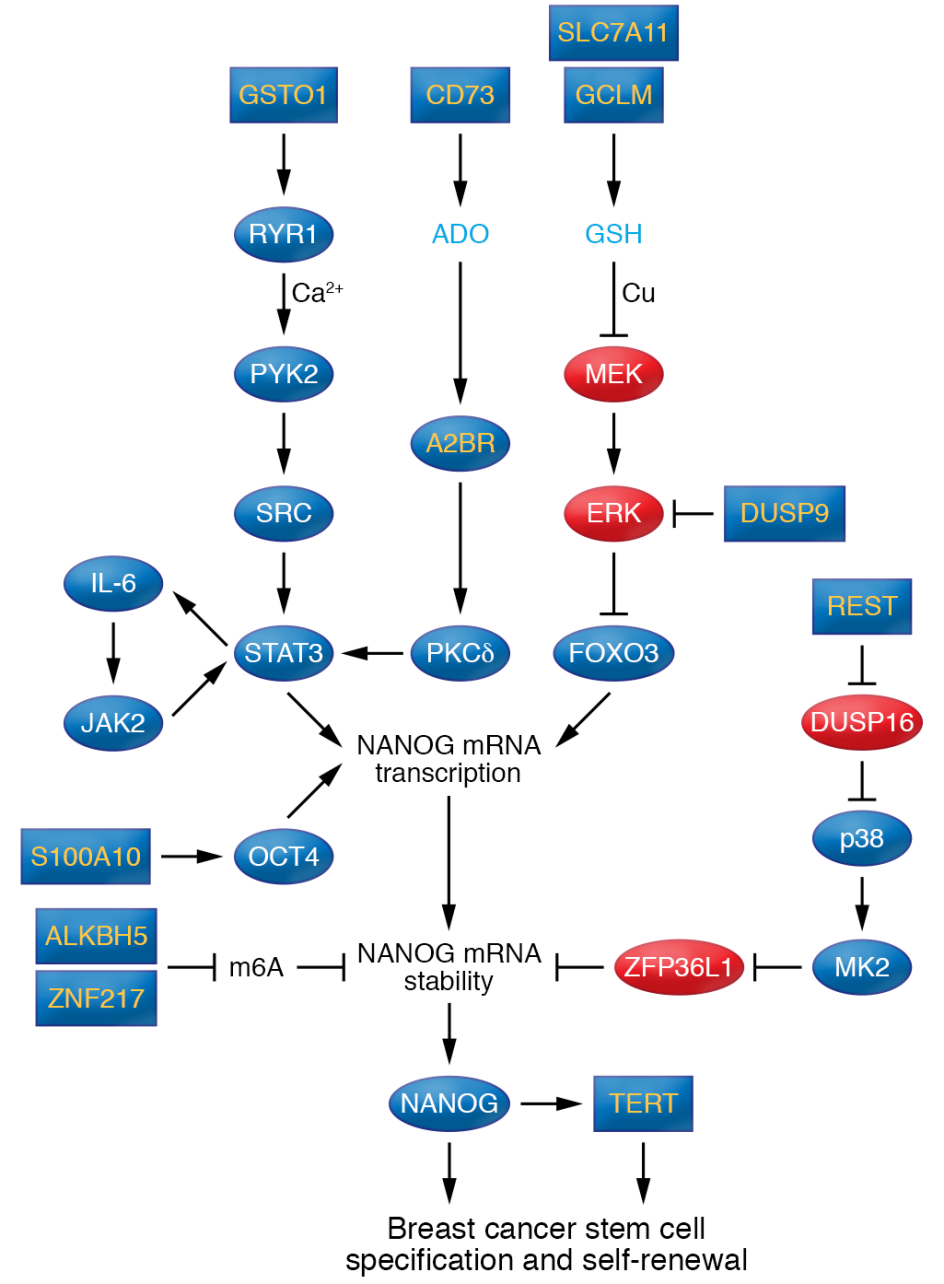
HIF target genes that induce NANOG expression and breast cancer stem cell specification. HIF target genes are denoted by yellow type in blue rectangles or ovals. Proteins that inhibit NANOG expression and breast cancer stem cell (BCSC) specification are shown in red ovals. Arrows and blocked arrows indicate positive and negative interactions, respectively. Ado, adenosine; Cu, copper; GSH, glutathione; m6A, methylation of adenosine residues in NANOG RNA.

In breast cancer, exposure of cells to 1% O_2_ for 72 hours is sufficient to double or triple the percentage of CSCs within the culture, and hypoxia induces CSC enrichment in vivo in a HIF-1–dependent manner (122, 123). Exposure of breast cancer cells to cytotoxic chemotherapy (e.g., carboplatin or paclitaxel) induces HIF activity leading to an increased percentage of CSCs among the surviving cells, both in vitro and in vivo (124), which may contribute to the common recurrence of triple-negative breast cancer (TNBC) after chemotherapy (125). Whereas the increased specification of breast CSCs in response to hypoxia is controlled solely by HIF-1 (122, 123), the response to chemotherapy is mediated by both HIF-1 and HIF-2 (124). Although HIF-1 may directly activate pluripotency gene expression in some cancer cells (126), a deep dive into the molecular mechanisms by which HIFs mediate increased breast CSC specification in response to hypoxia or chemotherapy has revealed multiple HIF target genes (Supplemental Table 2) that indirectly increase the synthesis or decrease the degradation of NANOG mRNA (Figure 2 and refs. 15, 127–132). HIFs indirectly activate NANOG transcription by increasing the activity of the transcription factors FOXO3 (128), OCT4 (130), and STAT3 (127, 129). In addition, HIF-1 induces WNT/β-catenin–dependent transcription in hypoxic breast CSCs (122) via expression of calreticulin (133). HIFs also mediate increased expression of genes encoding other transcription factors and epigenetic modifiers that are required for CSC specification (Supplemental Table 2).

In addition to the multiple mechanisms by which HIFs mediate increased NANOG expression, NANOG was found to function as a coactivator of HIF-1 in mediating increased transcription of the *TERT* gene in hypoxic breast CSCs (134). *TERT* encodes telomerase, the enzyme required for the maintenance of telomeres, which in turn are required for continued cell division and thus are required for infinite CSC self-renewal (135). Maintenance of the CSC phenotype is also dependent on maintenance of ROS homeostasis; hypoxia does not increase the percentage of breast CSCs when phosphoglycerate dehydrogenase (PHGDH), the first enzyme in the serine synthesis pathway (Figure 1), has been knocked down (63). Similarly, metastasis is dependent on breast CSCs: unlike control MDA-MB-231 cells, PHGDH-knockdown breast cancer cells do not metastasize from the mammary fat pad to the lungs, even though primary tumor growth is not impaired (63). In breast cancer patients, circulating tumor cells in peripheral blood with metastasis-initiating capability when injected into mice were found to express CD44, CD47, and MET (136), which are all encoded by HIF target genes (137–139).

These studies have revealed the plasticity of cancer cells with respect to the CSC phenotype, which is induced by intratumoral hypoxia. A striking implication is that many or perhaps every cancer cell can acquire CSC properties simply by residing in a hypoxic tumor microenvironment. Similarly, CSCs born in hypoxic niches may simply migrate less than 100 μm toward the well-oxygenated microenvironment near a blood vessel to switch off HIF activity and transition from the CSC to the transit-amplifying cell phenotype characterized by rapid cell division. Thus, it is likely that there is a selection for intratumoral hypoxia as the site of CSC specification. A corollary of this conclusion is that elimination of CSCs might be achieved by HIF inhibition or elimination of intratumoral hypoxia. Finally, it should be emphasized that hypoxia-induced breast CSC specification is mediated by HIF-1α only (38, 122), such that selective inhibition of HIF-2α (140), by inhibiting angiogenesis and increasing intratumoral hypoxia, might result in a counter-therapeutic increase in CSCs.

### Immune evasion.

**Figure 3:**
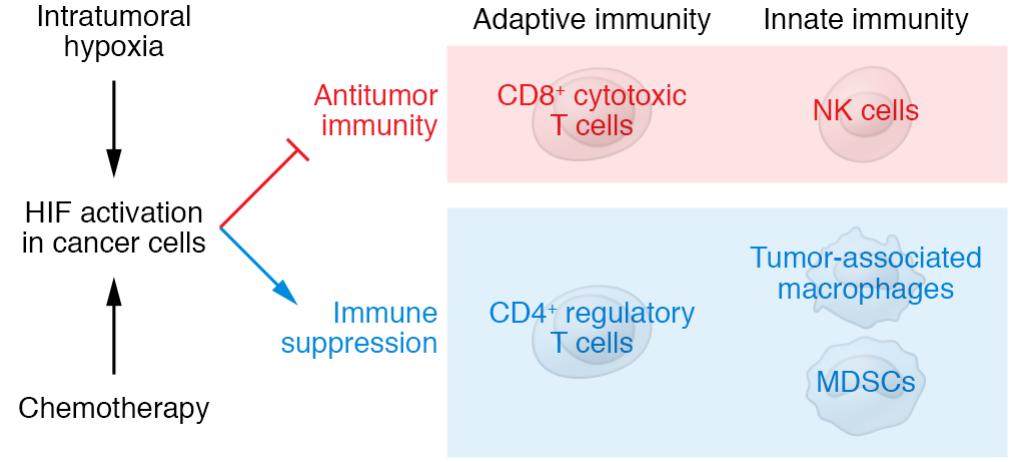
Cells of the adaptive and innate immune systems determine the balance between antitumor immunity and immune suppression. HIF activity in cancer cells inhibits antitumor immunity and promotes immune suppression.

In order for a cancer cell to form a metastatic focus, it must have CSC properties, and it must be able to evade killing by cells of the adaptive and innate immune systems. Cancer cells reprogram the tumor immune microenvironment to shift the balance from antitumor immunity to immunosuppression (141). Cytotoxic CD8^+^ T cells and natural killer (NK) cells are the major agents of adaptive and innate antitumor immunity, respectively. Cancer cells inhibit the activity of CD8^+^ T cells and stimulate the activity of regulatory T cells in order to evade killing by the adaptive immune system (Figure 3). Cancer cells also inhibit the activity of NK cells and recruit tumor-associated macrophages (TAMs) and myeloid-derived suppressor cells (MDSCs), components of the innate immune system that promote immunosuppression (141). A growing number of HIF target genes mediate this reprogramming of the tumor immune microenvironment (37–42). It is striking that many HIF target genes that mediate immune evasion also play important roles in mediating other critical aspects of cancer progression, such as angiogenesis, CSC specification, and metabolism (Supplemental Table 3).

Cancer cells are known to take up large quantities of glucose through the glucose transporter GLUT1 (encoded by the *SLC2A1* gene) and produce lactic acid (through the activity of LDHA and PDK1), either as a response to intratumoral hypoxia or driven in an O_2_-independent manner by genetic alterations (which is known as the Warburg phenomenon), and the lactate and H^+^ ions generated by LDHA are pumped out of the cancer cell by the monocarboxylate transporter MCT4 (encoded by the *SLC16A3* gene), the carbonic anhydrase CA9, and the sodium hydrogen exchanger NHE1 (encoded by the *SLC9A1* gene) (142–144). The resulting decrease in extracellular glucose and increase in extracellular lactate and H^+^ are all immunosuppressive (145–148). Thus, one of the classical features of advanced cancers, glycolytic metabolism, which previously was interpreted solely in terms of cancer cell energetics and proliferation, is now appreciated to play a critical role in the establishment of an immunosuppressive tumor microenvironment.

Hypoxia-induced expression of PDL1 (encoded by the *CD274* gene) and production of adenosine by cancer cells through the activity of CD73 (encoded by the *NT5E* gene) lead to immunosuppression (Supplemental Table 3) via binding to cognate receptors (PD-1 and adenosine receptor 2A, respectively) on NK and CD8^+^ T cells that results in exhaustion or apoptosis. However, the direct effect of hypoxia on T cells is less clear. Hypoxia has been reported to induce the expression of markers of T cell exhaustion, such as TIM3 and LAG3, as well as costimulatory receptors, such as 4-1BB, GITR, and OX40 (149). CD8^+^ T cells with increased HIF activity due to VHL conditional KO also express markers of tissue-resident memory T cells (CD69 and CD103) and have increased antitumor activity (150). However, it appears that the net effect of hypoxia in most tumors is immunosuppression, and administration of supplemental O_2_ (151) or a drug that is selectively toxic to hypoxic cells (152) is sufficient to increase the number of intratumoral T cells and the response to immunotherapy.

Women with TNBC are not eligible for targeted therapies and are treated with cytotoxic chemotherapy that often provides only a brief remission before recurrence, often in the form of metastatic disease. TNBC cells that survive cytotoxic chemotherapy, such as carboplatin or paclitaxel, stimulate an immunosuppressive tumor microenvironment with increased numbers of MDSCs and TAMs, decreased expression of NK and CD8^+^ T cells, and increased HIF activity that drives expression of CD73, PD-L1, and CD47, the latter of which protects cancer cells from phagocytosis by macrophages (153). Cytotoxic chemotherapy also induces HIF-dependent CSC specification (124), resulting in tumor-initiating cells that can evade both adaptive and innate immunity. Coadministration of the HIF inhibitor acriflavine with paclitaxel or carboplatin blocks the induction of an immunosuppressive tumor microenvironment (153). In melanoma and breast cancer cells, hypoxia induces HIF-dependent expression of BIRC2, which inhibits expression of CXCL9, thereby blocking recruitment of NK and CD8^+^ T cells to the tumor, leading to increased tumor growth and resistance to anti–PD-1 therapy (154).

Intratumoral hypoxia affects not only cancer cells but also the stromal cells within the tumor, most notably immune cells (39, 40). In contrast to the large body of data presented above indicating that HIF activity in cancer cells drives immune evasion, studies focused on the conditional KO of HIFs in immune cell populations have reported that HIFs play important cell-autonomous roles in CD4^+^ and CD8^+^ effector T cells, MDSCs, TAMs, Th17 cells, and NK cells; however, many of these studies have revealed distinct roles for HIF-1α versus HIF-2α, and distinct effects of loss of function in the same immune cell type in different tumor models (39). In recent studies using mouse models of breast cancer described above (153) and hepatocellular carcinoma described below, we have found that the net effect of pharmacologic inhibition of HIFs is to significantly increase antitumor immunity.

## Targeting HIFs for cancer therapy

A large number of chemical compounds have been shown to inhibit HIF activity in cancer cell lines (155). HIF inhibitors that have shown antitumor activity in mouse tumor models include acriflavine (47), digoxin (106), echinomycin (156), the HSP90 inhibitors 17-allylaminogeldanamycin (157) and ganetespib (158), 2-methoxyestradiol (159), PX-478 (160), and YC-1 (161). Many of these compounds are too toxic for use in humans as HIF inhibitors or have failed in clinical trials. Two compounds, PT2385 and PT2399, were shown to bind directly and selectively to HIF-2α and block its dimerization with HIF-1β, thereby inhibiting the expression of HIF-2 target genes and the growth of HIF-2–dependent RCC xenografts (162–164). Both compounds showed safety and activity against advanced RCC in phase I trials (165, 166). PT2399 (belzutifan) showed efficacy against RCC and other tumors in patients with von Hippel-Lindau syndrome in a phase III trial (140) and was recently approved by the FDA for this indication (167). RCC and other tumors in von Hippel-Lindau syndrome patients are outliers in that disease progression is often associated with loss of HIF-1α expression, which may be due in part to selection against HIF-1α–dependent inhibition of MYC activity (168). This stands in contrast to most cancer types, in which increased HIF-1α expression is associated with disease progression and patient mortality (Supplemental Table 1). The development of belzutifan is a major advance in the treatment of RCC, but preclinical studies revealed that some RCC cell line–derived tumors and patient-derived xenografts showed resistance to HIF-2 inhibitors (162, 164). One mouse model of RCC that was resistant to PT2385 responded to treatment with acriflavine (169), which binds to HIF-2α (and HIF-1α) at a site different from that of PT2385 (47, 170). There are multiple registrations for clinical trials involving belzutifan at ClinicalTrials.gov (Supplemental Table 4).

Hepatocellular carcinoma (HCC) is the most rapidly growing cause of cancer mortality in the United States with a 5-year survival rate of less than 12% (171). Nivolumab, an anti–PD-1 antibody, received FDA approval based on phase II clinical trial data, but the phase III trial did not meet its primary endpoint, and a phase III trial of pembrolizumab, another anti–PD-1 antibody, also failed as second-line therapy (172, 173). Liver cancers are characterized by severe intratumoral hypoxia with a median pO_2_ of 6 mmHg (0.9% O_2_) compared with 30 mmHg in normal liver tissue (174). Phosphatidylinositol-3-kinase and AKT signaling also contribute to increased HIF activity in HCC (175). Increased HIF-1α expression in the HCC diagnostic biopsy is associated with decreased disease-free and overall survival (176). In nude mice bearing Hep3B human HCC tumor xenografts, treatment with 32-134D, a novel HIF inhibitor, decreased the expression of HIF-1 and HIF-2 target genes and decreased tumor vascularization (177). In immunocompetent mice bearing Hepa1-6 mouse HCC tumors, combined treatment with 32-134D and anti–PD-1 antibody resulted in tumor eradication in 67% of the mice compared with 25% of the mice treated with anti–PD-1 alone (177). Treatment with 32-134D resulted in a significant increase in intratumoral NK and CD8^+^ T cells and a significant decrease in intratumoral MDSCs and TAMs, which were associated with increased expression of CXCL9 and CXCL10, the key chemokines for NK and T cell recruitment (177).

Whereas treatment of humans or mice with PT2385 or PT2399/belzutifan caused anemia due to inhibition of erythropoietin expression (140, 165, 166), treatment of mice with 32-134D did not affect plasma erythropoietin levels or red blood cell indices and had no effect on appearance, behavior, or body weight (177). HIFs play critical roles in vascular remodeling in response to ischemic cardiovascular disease (8), and treatment of mice with the HIF inhibitor 2-methoxyestradiol inhibited recovery of blood flow and increased tissue injury after femoral artery ligation (21). It is therefore possible that HIF inhibitors might have adverse effects in patients with severe coronary or peripheral artery disease. However, the observation that 32-134D does not affect blood erythropoietin levels (177) suggests that it might have a selective inhibitory effect in HCC cells. Clinical trials will determine whether a therapeutic window exists for the safe and efficacious use of combined HIF-1/HIF-2 inhibitors for cancer therapy. A recent review summarized the experimental data supporting the concept that HIF-1 and HIF-2 play complementary roles in many cancers and that targeting both HIF-1 and HIF-2 for inhibition will provide greater therapeutic benefit than targeting either one of them alone (39). Given the requirement for HIF expression in many cancer types, the extraordinarily high levels of expression in comparison with normal tissues, and the limited evidence of toxicity in mouse models, it seems likely that HIF inhibitors will be valuable new weapons with which to fight cancer.

## Supplementary Material

Supplemental data

## Version 1 06/01/2022

Electronic publication
