# Supplementary material for: Hypoxia-inducible factors: cancer progression and clinical translation
Source: J Clin Invest. 2022 Jun 1;132(11):e159839. doi: 10.1172/JCI159839 (PMC9151701; doi:10.1172/JCI159839)
Supplement: Supplemental data [file jci-132-159839-s171.pdf]

Supplemental Table 1. Association of HIF expression and patient mortality.

Cancer type	HIF-1/2α	References
Bladder	HIF-1α	1, 2
Brain	HIF-1α	3-8
	HIF-2α	9
Breast	HIF-1α	10-22
	HIF-2α	23, 24
Cervix	HIF-1α	25-29
Chondrosarcoma	HIF-2α	30
Colon	HIF-1α	31-35
Endometrial	HIF-1α	36, 37
Esophagus	HIF-1α	38-41
Gastric	HIF-1α	42-48
GIST, stomach	HIF-1α	49
Gynecological	HIF-1α	50
Head and neck-SCC	HIF-1α	51, 52
	HIF-2α	53
Hepatocellular	HIF-1α	54-58
	HIF-2α	59
Laryngeal	HIF-1α	60
Leukemia	HIF-1α	61-63
Liver	HIF-1α	64
Lung	HIF-1α	65-72
	HIF-2α	73, 74
Melanoma	HIF-2α	75
MDS	HIF-1α	76
Oral SCC	HIF-1α	77-82
Osteosarcoma	HIF-1α	83
Ovarian	HIF-1α	84-88
Pancreas	HIF-1α	89-94
	HIF-2α	93
Prostate	HIF-1α	95, 96
	HIF-2α	95
Rectal	HIF-1α	97-99
Renal	HIF-1α, HIF-2α	100
Urinary tract	HIF-1α	101

References

1. Theodoropoulos VE, et al. Hypoxia-inducible factor 1α expression correlates with angiogenesis and unfavorable prognosis in bladder cancer. *Eur Urol*. 2004;46(2):200-208.
2. Theodoropoulos VE, et al. Evaluation of hypoxia-inducible factor 1α overexpression as a predictor of tumor recurrence and progression in superficial urothelial bladder carcinoma. *BJU Int.* 2005;95(3):425-431.
3. Birner P, et al. Expression of hypoxia-inducible factor–1α in oligodendrogliomas: its impact on prognosis and on neoangiogenesis. *Cancer*. 2001;92(1):165-171.
4. Jensen R, Lee J. Predicting outcomes of patients with intracranial meningiomas using molecular markers of hypoxia, vascularity, and proliferation. *Neurosurgery*. 2012;71(1):146-156.
5. Ji X, et al. Correlation of Nrf2 and HIF-1α in glioblastoma and their relationships to clinicopathologic features and survival. *Neurol Res*. 2013;35(10):1044-1050.
6. Korkolopoulou P, et al. Hypoxia-inducible factor 1α/vascular endothelial growth factor axis in astrocytomas. Associations with microvessel morphometry, proliferation and prognosis. *Neuropathol Appl Neurobiol*. 2004;30(3):267-278.

7. Potharaju M, et al. Clinicopathological analysis of HIF-1α and TERT on survival outcome in glioblastoma patients: a prospective, single institution study. *J Cancer*. 2019;10(11):2397-2406.
8. Sfifou F, et al. Immunohistochemical expression of HIF-1α, IDH1 and TP53: prognostic profile of Moroccan patients with diffuse glioma. *J Chem Neuroanat*. 2021;119:102056.
9. Li Z, et al. Hypoxia-inducible factors regulate tumorigenic capacity of glioma stem cells. *Cancer Cell*. 2009;15(6):501-513.
10. Bos R, et al. Levels of hypoxia-inducible factor-1α independently predict prognosis in patients with lymph node negative breast carcinoma. *Cancer*. 2003;97(6):1573-1581.
11. Dales JP, et al. Overexpression of hypoxia-inducible factor HIF-1α predicts early relapse in breast cancer: retrospective study in a series of 745 patients. *Int J Cancer*. 2005;116(5):734-739.
12. Giatromanolaki A, et al. c-erbB-2 related aggressiveness in breast cancer is hypoxia inducible factor-1α dependent. *Clin Cancer Res*. 2004;10(23):7972-7977.
13. Gruber G, et al. Hypoxia-inducible factor 1α in high-risk breast cancer: an independent prognostic parameter? *Breast Cancer Res*. 2004;6(3):R191-198.
14. Jin MS, et al. Overexpression of HIF1α and CAXI predicts poor outcome in early-stage triple negative breast cancer. *Virchows Arch*. 2016;469(2):183-190.
15. Kaya AO, et al. Hypoxia inducible factor-1α and carbonic anhydrase IX overexpression are associated with poor survival in breast cancer patients. *J BUON*. 2012;17(4):663-668.
16. Kronblad A, et al. Hypoxia inducible factor-1α is a prognostic marker in premenopausal patients with intermediate to highly differentiated breast cancer but not a predictive marker for tamoxifen response. *Int J Cancer*. 2006;118(10):2609-2616.
17. Ni X, et al. Hypoxia-induced factor-1α upregulates vascular endothelial growth factor C to promote lymphangiogenesis and angiogenesis in breast cancer patients. *J Biomed Res*. 2013;27(6):478-485.
18. Schindl M, et al. Overexpression of hypoxia-inducible factor 1α is associated with an unfavorable prognosis in lymph node-positive breast cancer. *Clin Cancer Res*. 2002;8(6):1831-1837.
19. Trastour C, et al. HIF-1α and CA IX staining in invasive breast carcinomas: prognosis and treatment outcome. *Int J Cancer*. 2007;120(7):1451-1458.
20. Vleugel MM, et al. Differential prognostic impact of hypoxia induced and diffuse HIF-1α expression in invasive breast cancer. *J Clin Pathol*. 2005;58(2):172-177.
21. Yamamoto Y, et al. Hypoxia-inducible factor 1α is closely linked to an aggressive phenotype in breast cancer. *Breast Cancer Res Treat*. 2008;110(3):465-475.
22. Yan M, et al. BRCA1 tumors correlate with a HIF-1α phenotype and have a poor prognosis through modulation of hydroxylase enzyme profile expression. *Br J Cancer.* 2009;101(7): 1168-1174.
23. Helczynska K, et al. Hypoxia-inducible factor-2α correlates to distant recurrence and poor outcome in invasive breast cancer. *Cancer Res.* 2008;68(22):9212-9220.
24. Leek RD, et al. Relation of hypoxia-inducible factor-2α (HIF-2α) expression in tumor-infiltrative macrophages to tumor angiogenesis and the oxidative thymidine phosphorylase pathway in human breast cancer. *Cancer Res*. 2002;62(5):1326-1329.
25. Bachtiary B, et al. Overexpression of hypoxia-inducible factor 1α indicates diminished response to radiotherapy and unfavorable prognosis in patients receiving radical radiotherapy for cervical cancer. *Clin Cancer Res*. 2003;9(6):2234-2240.
26. Birner P, et al. Overexpression of hypoxia-inducible factor 1α is a marker for an unfavorable prognosis in early-stage invasive cervical cancer. *Cancer Res*. 2000;60(17):4693-4696.
27. Burri P, et al. Significant correlation of hypoxia-inducible factor-1α with treatment outcome in cervical cancer treated with radical radiotherapy. *Int J of Radiat Oncol Biol Phys*. 2003;56(2): 494-501.
28. Huang M, et al. Overexpression of hypoxia-inducible factor-1α is a predictor of poor prognosis in cervical cancer: A clinicopathologic study and a meta-analysis. *Int J of Gynecol Cancer*. 2014;24(6):1054-1064.
29. Kim BW, et al. Prognostic assessment of hypoxia and metabolic markers in cervical cancer using automated digital image analysis of immunohistochemistry. *J Transl Med*. 2013;11:185.
30. Chen C, et al. Increased levels of hypoxia-inducible factor-1α are associated with Bcl-xL expression, tumor apoptosis, and clinical outcome in chondrosarcoma. *J Orthop Res*. 2011; 29(1):143-51.
31. Baba Y, et al. HIF1A overexpression is associated with poor prognosis in a cohort of 731 colorectal cancers. *Am J Pathol*. 2010;176(5):2292-2301.
32. Cao D, et al. Expression of HIF-1α and VEGF in colorectal cancer: association with clinical outcomes and prognostic implications. *BMC Cancer*. 2009;9:432
33. Rajaganeshan R, et al. The role of hypoxia in recurrence following resection of Dukes' B colorectal cancer. *Int J Colorectal Dis*. 2008;23(11):1049-1055.

34. Schmitz KJ, et al. Combined analysis of hypoxia-inducible factor 1α and metallothionein indicates an aggressive subtype of colorectal carcinoma. *Int J Colorectal Dis*. 2009;24(11): 1287-1296.
35. Tang Y, et al. Hypoxic tumor microenvironment activates GLI2 via HIF-1α and TGF-β2 to promote chemoresistance in colorectal cancer. *Proc Natl Acad Sci U S A*. 2018;115(26): E5990-E5999.
36. Seeber L, et al. Hypoxia-inducible factor-1 as a therapeutic target in endometrial cancer management. *Obstet Gynecol Int*. 2010;2010:580971.
37. Sivridis E, et al. Association of hypoxia-inducible factors 1α and 2α with activated angiogenic pathways and prognosis in patients with endometrial carcinoma. *Cancer*. 2002;95(5):1055-1063.
38. Kurokawa T, et al. Overexpression of hypoxia-inducible-factor 1α (HIF-1α) in esophageal squamous cell carcinoma correlates with lymph node metastasis and pathologic stage. *Br J Cancer*. 2003;89(6):1042-1047.
39. Ping W, et al. Clinicopathological and prognostic significance of hypoxia-inducible factor-1α in esophageal squamous cell carcinoma: a meta-analysis. *Tumour Biol*. 2014;35(5):4401-4409.
40. Tzao C, et al. Expression of hypoxia-inducible factor (HIF)-1α and vascular endothelial growth factor (VEGF)-D as outcome predictors in resected esophageal squamous cell carcinoma. *Dis Markers*. 2008;25(3):141-148.
41. Zhang L, et al. Increased HIF-1α expression in tumor cells and lymphocytes of tumor microenvironments predicts unfavorable survival in esophageal squamous cell carcinoma patients. *Int J Clin Exp Pathol*. 2014;7(7):3887-3897.
42. Chen J, et al. Clinical and prognostic significance of HIF-1α, PTEN, CD44v6, and survivin for gastric cancer: a meta-analysis. *PLoS One*. 2014;9(3):e91842.
43. Griffiths EA, et al. Hypoxia-inducible factor-1α expression in the gastric carcinogenesis sequence and its prognostic role in gastric and gastro-esophageal adenocarcinomas. *Br J Cancer*. 2007;96(1):95-103.
44. Isobe T, et al. Clinicopathological significance of hypoxia-inducible factor-1α (HIF-1α) expression in gastric cancer. *Int J Clin Oncol*. 2013;18(2):293-304.
45. Lin S, et al. Meta-analysis of immunohistochemical expression of hypoxia inducible factor-1α as a prognostic role in gastric cancer. *World J Gastroenterol*. 2014;20(4):1107-1113.
46. Qiu MZ, et al. Expressions of hypoxia-inducible factor-1α and hexokinase-II in gastric adenocarcinoma: the impact on prognosis and correlation to clinicopathologic features. *Tumour Biol*. 2011;32(1):159-166.
47. Zhang WJ, et al. Hypoxia-inducible factor-1α correlates with tumor-associated macrophages infiltration, influences survival of gastric cancer patients. *J Cancer.* 2017;8(10):1818-1825.
48. Zhang J, et al. Prognostic value of hypoxia-inducible factor-1α and prolyl 4-hydroxylase β polypeptide overexpression in gastric cancer. *World J Gastroenterol.* 2018;24(22):2381-2391.
49. Takahashi R, et al. Hypoxia-inducible factor-1α expression and angiogenesis in gastrointestinal stromal tumor of the stomach. *Oncol Rep*. 2003;10(4):797-802.
50. Jin Y, et al. Clinicopathological characteristics of gynecological cancer associated with hypoxia-inducible factor 1α expression: a meta-analysis including 6,612 subjects. *PLoS One*. 2015;10(5):e0127229.
51. Beasley NJ, et al. Hypoxia-inducible factors HIF-1α and HIF-2α in head and neck cancer: relationship to tumor biology and treatment outcome in surgically resected patients. *Cancer Res*. 2002;62(9):2493-2497.
52. Kappler M, et al. Immunohistochemical detection of HIF-1α and CAIX in advanced head-and-neck cancer. Prognostic role and correlation with tumor markers and tumor oxygenation parameters. *Strahlenther Onkol*. 2008;184(8):393-399.
53. Koukourakis MI, et al. Hypoxia-inducible factor (HIF1A and HIF2A), angiogenesis, and chemoradiotherapy outcome of squamous cell head-and-neck cancer. *Int J Radiat Oncol Biol Phys*. 2002;53(5):1192-1202.
54. Cao S, et al. Protein expression of hypoxia-inducible factor-1α and hepatocellular carcinoma: a systematic review with meta-analysis. *Clin Res Hepatol Gastroenterol*. 2014;38(5):598-603.
55. Dai Y, et al. Integrated analysis of hypoxia-induced miR-210 signature as a potential prognostic biomarker of hepatocellular carcinoma: a study based on The Cancer Genome Atlas. *J Zhejiang Univ Sci B*. 2019;20(11):928-932.
56. Xia L, et al. The TNF-α/ROS/HIF-1-induced upregulation of FoxMI Expression promotes HCC proliferation and resistance to apoptosis. *Carcinogenesis*. 2012;33(11):2250-2259.
57. Xiang ZL, et al. The expression of HIF-1α in primary hepatocellular carcinoma and its correlation with radiotherapy response and clinical outcome. *Mol Biol Rep*. 2012;39(2):2021-2029.
58. Zheng SS, et al. Prognostic significance of HIF-1α expression in hepatocellular carcinoma: a meta-analysis. *PLoS One*. 2013;8(6):e65753.
59. Bangoura G, et al. Prognostic significance of HIF-2α/EPAS1 expression in hepatocellular carcinoma. *World J Gastroenterol*. 2007;13(23):3176-3182.

60. Schrijvers ML, et al. Overexpression of intrinsic hypoxia markers HIF-1α and CA-IX predict for local recurrence in stage T1-T2 glottic laryngeal carcinoma treated with radiotherapy. *Int J Radiat Oncol Biol Phys*. 2008;72(1):161-169.
61. Deeb G, et al. Hypoxia-inducible factor-1α protein expression is associated with poor survival in normal karyotype adult acute myeloid leukemia. *Leuk Res*. 2011;35(5):579-584.
62. Frolova O, et al. Regulation of HIF-1α signaling and chemoresistance in acute lymphocytic leukemia under hypoxic conditions of the bone marrow microenvironment. *Cancer Biol Ther*. 2012;13(10):858-870.
63. Wellmann S, et al. Activation of the HIF pathway in childhood ALL, prognostic implications of VEGF. *Leukemia*. 2004;18(5):926-933.
64. Morine Y, et al. Hypoxia inducible factor expression in intrahepatic cholangiocarcinoma. *Hepatogastroenterology*. 2011;58(110-111):1439-1444.
65. Hung JJ, et al. Prognostic significance of hypoxia-inducible factor-1α, TWIST1 and Snail expression in resectable non-small cell lung cancer. *Thorax*. 2009;64(12):1082-1089.
66. Ioannou M, et al. Hypoxia inducible factor-1α and vascular endothelial growth factor in biopsies of small cell lung carcinoma. *Lung*. 2009;187(5):321-329.
67. Koh YW, et al. PD-L1 protein expression in non-small-cell lung cancer and its relationship with the hypoxia-related signaling pathways: A study based on immunohistochemistry and RNA sequencing data. *Lung Cancer*. 2019;129:41-47.
68. Lin CS, et al. Independent prognostic value of hypoxia-inducible factor 1α expression in small cell lung cancer. *Int J Med Sci*. 2017;14(8):785-790.
69. Park S, et al. Prognostic implications of hypoxia-inducible factor-1α in epidermal growth factor receptor-negative non-small cell lung cancer. *Lung Cancer*. 2011;72(1):100-107.
70. Swinson DE, et al. Hypoxia-inducible factor-1α in non-small cell lung cancer: relation to growth factor, protease and apoptosis pathways. *Int J Cancer*. 2004;111(1):43-50.
71. Volm M, Koomägi R. Hypoxia-inducible factor (HIF-1) and its relationship to apoptosis and proliferation in lung cancer. *Anticancer Res*. 2000;20(3A):1527-1533.
72. Wang Q, et al. Prognosis value of HIF-1α expression in patients with non-small cell lung cancer. *Gene*. 2014;541(2):69-74.
73. Gao ZJ, et al. HIF-2α not HIF-1α overexpression confers poor prognosis in non–small cell lung cancer. *Tumour Biol*. 2017;39(6):1010428317709637.
74. Giatromanolaki A, et al. Relation of hypoxia inducible factor 1α and 2α in operable non-small cell lung cancer to angiogenic/molecular profile of tumors and survival. *Br J Cancer.* 2001; 85(6):881-890.
75. Giatromanolaki A, et al. Hypoxia-inducible factors 1α and 2α are related to vascular endothelial growth factor expression and a poorer prognosis in nodular malignant melanomas of the skin. *Melanoma Res*. 2003;13(5):493-501.
76. Tong H, et al. Hypoxia-inducible factor-1α expression indicates poor prognosis in myelodysplastic syndromes. *Leuk Lymphoma*. 2012;53(12):2412-2418.
77. Dunkel J, et al. Prognostic markers in stage I oral cavity squamous cell carcinoma. *Laryngoscope*. 2013;123(10):2435-2441.
78. Eckert AW, et al. Co-expression of HIF-1α and CAIX is associated with poor prognosis in oral squamous cell carcinoma patients. *J Oral Pathol Med*. 2010;39(4):313-317.
79. Fillies T, et al. HIF-1α overexpression indicates a good prognosis in early-stage squamous cell carcinomas of the oral floor. *BMC Cancer*. 2005;5:84.
80. Liang X, et al. Hypoxia-inducible factor-1α, in association with TWIST2 and SNIP1, is a critical prognostic factor in patients with tongue squamous cell carcinoma. *Oral Oncol*. 2011; 47(2):92-97.
81. Santos MD, et al. HIF-1α expression predicts survival of patients with squamous cell carcinoma of the oral cavity. *PLoS One*. 2012;7(9):e45228.
82. Zhu GQ, et al. Hypoxia inducible factor 1α and hypoxia inducible factor 2α play distinct and functionally overlapping roles in oral squamous cell carcinoma. *Clin Cancer Res*. 2010; 16(19):4732-4741.
83. Chen Y et al. Predicting chemosensitivity in osteosarcoma prior to chemotherapy: An investigational study of biomarkers with immunohistochemistry. *Oncol Lett*. 2012;3(5):1011-1016.
84. Birner P, et al. Expression of hypoxia-inducible factor 1α in epithelial ovarian tumors: its impact on prognosis and on response to chemotherapy. *Clin Cancer Res*. 2001;7(6):1661-1668.
85. Daponte A, et al. Prognostic significance of hypoxia-inducible factor 1α (HIF-1α) expression in serous ovarian cancer: an immunohistochemical study. *BMC Cancer*. 2008;8:335.
86. Jin Y, et al. Pathological and prognostic significance of hypoxia-inducible factor 1α expression in epithelial ovarian cancer: a meta-analysis. *Tumour Biol*. 2014;35(8):8149-8159.

87. Osada R, et al. Expression of hypoxia-inducible factor 1α, hypoxia-inducible factor 2α, and von Hippel–Lindau protein in epithelial ovarian neoplasms and allelic loss of von Hippel-Lindau gene: nuclear expression of hypoxia-inducible factor 1α is an independent prognostic factor in ovarian carcinoma. *Hum Pathol*. 2007;38(9):1310-1320.
88. Shen W, et al. Expression levels of PTEN, HIF-1α, and VEGF as prognostic factors in ovarian cancer. *Eur Rev Med Pharmacol Sci*. 2017;21(11):2596-2603.
89. Hoffmann AC, et al. High expression of HIF-1α is a predictor of clinical outcome in patients with pancreatic ductal adenocarcinomas and correlated to PDGFA, VEGF, and bFGF. *Neoplasia*. 2008;10(7):674-679.
90. Matsuo Y, et al. Hypoxia-inducible factor 1α plays a pivotal role in hepatic metastasis of pancreatic cancer: an immunohistochemical study. *J Hepatobiliary Pancreat Sci*. 2014; 21(2):105-112.
91. Qin R, et al. Combining clinicopathological predictors and molecular biomarkers in the oncogenic K-RAS/Ki67/HIF-1α pathway to predict survival in resectable pancreatic cancer. *Br J Cancer*. 2015;112(3):514-522.
92. Sun HC, et al. Expression of hypoxia-inducible factor-1α and associated proteins in pancreatic ductal adenocarcinoma and their impact on prognosis. *Int J Oncol*. 2007;30(6):1359-1367.
93. Wang M, et al. Expression and significance of HIF-1α and HIF-2α in pancreatic cancer. *J Huazhong Univ Sci Technolog Med Sci*. 2015;35(6):874-879.
94. Ye LY, et al. Hypoxia-inducible factor 1α expression and its clinical significance in pancreatic cancer: a meta-analysis. *Pancreatology*. 2014;14(5):391-397.
95. Nanni S, et al. Endothelial NOS, estrogen receptor β, and HIFs cooperate in the activation of a prognostic transcriptional pattern in aggressive human prostate cancer. *J Clin Invest.* 2009; 119(5):1093-1108.
96. Ranasinghe WK, et al. The role of hypoxia-inducible factor 1α in determining the properties of castrate-resistant prostate cancers. *PLoS One*. 2013;8(1):e54251.
97. Lin S, et al. Thymidine phosphorylase and hypoxia-inducible factor 1α expression in clinical stage II/III rectal cancer: association with response to neoadjuvant chemoradiation therapy and prognosis. *Int J Clin Exp Pathol.* 2015;8(9):10680-10688.
98. Lu X, et al. Clinical significance of immunohistochemical expression of hypoxia-inducible factor–1α as a prognostic marker in rectal adenocarcinoma. *Clin Colorectal Cancer.* 2006; 5(5):350-353.
99. Rasheed S, et al. Hypoxia-inducible factor-1α and -2α are expressed in most rectal cancers but only hypoxia-inducible factor-1α is associated with prognosis. *Br J Cancer*. 2009;100(10): 1666-1673.
100. Fan Y, et al. Prognostic significance of hypoxia-inducible factor expression in renal cell carcinoma: a PRISMA-compliant systematic review and meta-analysis. *Medicine (Baltimore)*. 2015;94(38):e1646.
101. Nakanishi K, et al. Expression of hypoxia-inducible factor-1α protein predicts survival in patients with transitional cell carcinoma of the upper urinary tract. *Clin Cancer Res*. 2005; 11(7):2583-2590.

Supplemental Table 2. Selected HIF target genes involved in metastasis.

Role in metastasis	*Type of encoded protein*	*Gene name*	*References*
Epithelial-mesenchymal transition (EMT) and tissue invasion	Transcription factor	SNAI1 (SNAIL)	1-2
		SNAI2 (SLUG)	3
		TWIST1, ZEB1, ZEB2	4-7
		TCF3	6
		EWS-FLI1	8
		SMAD7	9
		HEF1 (NEDD9)	10
	Receptor tyrosine kinase	AXL	11
		MET	12-14
	Transmembrane glycoprotein	CDCP1	15
		CD44	16, 17
	ECM-modifying enzyme	P4HA1, P4HA2	18-20
		PLOD2	21
		ADAM12	22
	Scaffold protein	AKAP12	23
	Small GTPase	RAB22A	24
	G-protein coupled receptor	CXCR3, CXCR4	25, 26
		CXCR6, CXCR7	27, 28
		CCR1, CCR5, CCR7	25, 29, 30
	Chemokine/ cytokine	CSF1, CXCL16	25
		HMGB1,	31
		CXCL8 (IL8)	32
Intravasation/circulation/ extravasation/metastasis	Histone modifier	JMJD2B, JMJD2C	33, 34
	Membrane protein	PLAUR (uPAR)	35, 36
		ITGA5	37
		AEG1 (MTDH)	38
		C1QBP	39
		ANGTPL4, L1CAM	40
		CD47	41-43
		CD73	41, 44
		CAV1	45
	ECM modifier	MMP1, MMP2	36, 46, 47
	Metabolic enzyme	CKB	48
		GLS1	49
	Receptor / Signaling	RHOA, ROCK1	50
		TRKB (NTRK2)	51, 52
		PTGS2 (COX2)	53, 54
	Growth factor	PDGFB	55
		PGF, VEGFA	25, 56
Pre-metastatic niche formation	ECM-modifying enzyme	LOX	57-59
		LOXL2, LOXL4	58, 59
	S100 protein	S100A8, S100A9	60, 61
Cancer stem cell specification and maintenance	Transporter	ABCB1 (MDR1)	62
		ABCG2 (BCRP)	63
		SLC7A11	64
	Enzyme	ALKBH5	65, 66
		DUSP9	67
		GSTO1	68
		PHGDH	69
		SIAH1	70
		TERT	71
		GCLM	72

	Membrane receptor	CD47<br>A2BR<br>CALR	73<br>74<br>75
	S100 protein	S100A10	76
	Interleukin	IL6, IL8	32, 62
	Transcription factor	HES1, HEY1<br>REST<br>SNAI1<br>WWTR1<br>ZNF217	77<br>78<br>1-2<br>70<br>66, 79
	Epigenetic modifier	KDM4C (JMJD2C)<br>KDM6B (JMJD3)<br>TET1, TET3	34<br>80<br>81, 82

References

1. Luo D, Wang J, Li J, Post M. Mouse snail is a target gene for HIF. *Mol Cancer Res.* 2011;9(2): 234-245.
2. Yang SW, et al. HIF-1α induces the epithelial-mesenchymal transition in gastric cancer stem cells through the Snail pathway. *Oncotarget.* 2017;8(6):9535-9545.
3. Regulation of membrane-type 4 matrix metalloproteinase by SLUG contributes to hypoxia-mediated metastasis. *Neoplasia*. 2009;11(12):1371-1382.
4. Yang MH, et al. Direct regulation of TWIST by HIF-1α promotes metastasis. *Nat Cell Biol*. 2008;10(3):295-305.
5. Tsai CC, et al. Hypoxia inhibits senescence and maintains mesenchymal stem cell properties through down-regulation of E2A-p21 by HIF-TWIST. *Blood*. 2011;117(2):459-469.
6. Krishnamachary B, et al. Hypoxia-inducible factor-1-dependent repression of E-cadherin in von Hippel-Lindau tumor suppressor-null renal cell carcinoma mediated by TCF3, ZFHX1A, and ZFHX1B. *Cancer Res* 2006;66(5):2725-2731.
7. Depner C, et al. EphrinB2 repression through ZEB2 mediates tumor invasion and anti-angiogenic resistance. *Nat Commun*. 2016;7:12329.
8. Aryee DN, et al. Hypoxia modulates EWS-FLI1 transcriptional signature and enhances the malignant properties of Ewing's sarcoma cells in vitro. *Cancer Res*. 2010;70(10):4015-4023.
9. Heikkinen PT, et al. Hypoxic conversion of SMAD7 function from an inhibitor into a promoter of cell invasion. *Cancer Res.* 2010;70(14):5984-5993.
10. Kim SH, et al. Human enhancer of filamentation 1 is a mediator of hypoxia-inducible factor-1a-mediated migration in colorectal carcinoma cells. *Cancer Res.* 2010;70(10):4054-4063.
11. Rankin EB, et al. Direct regulation of GAS6/AXL signaling by HIF promotes renal metastasis through SRC and MET. *Proc Natl Acad Sci U S A*. 2014;111(37):13373-13378.
12. Hara S, et al. Hypoxia enhances c-Met/HGF receptor expression and signaling by activating HIF-1a in human salivary gland cancer cells. *Oral Oncol*. 2006;42(6):593-598.
13. Pennacchietti S, et al. Hypoxia promotes invasive growth by transcriptional activation of the met protooncogene. *Cancer Cell*. 2003;3(4):347-361.
14. Schelter F, et al. Tissue inhibitor of metalloproteinases-1-induced scattered liver metastasis is mediated by hypoxia-inducible factor-1α. *Clin Exp Metastasis*. 2011;28(2):91-99.
15. Cao M, et al. HIF-2α regulates CDCP1 to promote PKCδ-mediated migration in hepatocellular carcinoma. *Tumour Biol*. 2016;37(2):1651-1662.
16. Bai J, et al. HIF-2α regulates CD44 to promote cancer stem cell activation in triple-negative breast cancer *via*PI3K/AKT/mTOR signaling. *World J Stem Cells*. 2020;12(1):87-99.
17. Krishnamachary B, et al. Hypoxia regulates CD44 and its variant isoforms through HIF-1α in triple negative breast cancer. *PLoS One*. 2012;7(8):e44078.
18. Takahashi Y, Takahashi S, Shiga Y, Yoshimi T, Miura T. Hypoxic induction of prolyl 4-hydroxylase α(I) in cultured cells. *J Biol Chem.* 2000;275(19):14139-14146.
19. Gilkes DM, et al. Collagen prolyl hydroxylases are essential for breast cancer metastasis. *Cancer Res.* 2013;73(11):3285-3296.
20. Gilkes DM, et al. Hypoxia-inducible factor 1 (HIF-1) promotes extracellular matrix remodeling under hypoxic conditions by inducing P4HA1, P4HA2, and PLOD2 expression in fibroblasts. *J Biol Chem*. 2013;288(15):10819-10829.

21. Gilkes DM, et al. Procollagen lysyl hydroxylase 2 is essential for hypoxia-induced breast cancer metastasis. *Mol Cancer Res*. 2013;11(5):456-466.
22. Wang R, et al. Hypoxia-inducible factor-dependent ADAM12 expression mediates breast cancer invasion and metastasis. *Proc Natl Acad Sci U S A*. 2021;118(19):e2020490118.
23. Finger EC, et al. Hypoxic induction of AKAP12 variant 2 shifts PKA-mediated protein phosphorylation to enhance migration and metastasis of melanoma cells. *Proc Natl Acad Sci U S A*. 2015;112(14):4441-4446.
24. Wang T, et al. Hypoxia-inducible factors and RAB22A mediate formation of microvesicles that stimulate breast cancer invasion and metastasis. *Proc Natl Acad Sci U S A*. 2014;111(31):E3234-E3242.
25. Chaturvedi P, et al. Hypoxia-inducible factor-dependent signaling between triple-negative breast cancer cells and mesenchymal stem cells promotes macrophage recruitment. *Proc Natl Acad Sci U S A*. 2014;111(20):E2120-E2129.
26. Schioppa T, et al. Regulation of the chemokine receptor CXCR4 by hypoxia. *J Exp Med.* 2003;198(9):1391-1402.
27. Lin S, et al. Chemokine C-X-C motif receptor 6 contributes to cell migration during hypoxia. *Cancer Lett*. 2009;279(1):108-117.
28. Liu H, et al. Hypoxic preconditioning advances CXCR4 and CXCR7 expression by activating HIF-1α in MSCs. *Biochem Biophys Res Commun*. 2010;401(4):509-515.
29. Wilson JL, et al. Endothelins induce CCR7 expression by breast tumor cells via endothelin receptor A and hypoxia-inducible factor-1. *Cancer Res*. 2006;66(24):11802-11807.
30. Zhu Y, et al. C-C chemokine receptor type 1 mediates osteopontin-promoted metastasis in hepatocellular carcinoma. *Cancer Sci*. 2018;109(3):710-723.
31. Jiang J, et al. Hypoxia-induced HMGB1 expression of HCC promotes tumor invasiveness and metastasis via regulating macrophage-derived IL-6. *Exp Cell Res*. 2018;367(1):81-88.
32. Feng W, et al. HIF-1α promotes the migration and invasion of hepatocellular carcinoma cells via the IL-8-NF-κB axis. *Cell Mol Biol Lett*. 2018;23:26.
33. Fu L, et al. HIF-1α-induced histone demethylase JMJD2B contributes to the malignant phenotype of colorectal cancer cells via an epigenetic mechanism. *Carcinogenesis*. 2012; 33(9):1664-1673.
34. Luo W, Chang R, Zhong J, Pandey A, Semenza GL. Histone demethylase JMJD2C is a coactivator for hypoxia-inducible factor 1 that is required for breast cancer progression. *Proc Natl Acad Sci U S A.* 2012;109(49):E3367-E3376.
35. Büchler P, et al. Transcriptional regulation of urokinase-type plasminogen activator receptor by hypoxia-inducible factor 1 is crucial for invasion of pancreatic and liver cancer. *Neoplasia*. 2009; 11(2):196-206.
36. Krishnamachary B, et al. Regulation of colon carcinoma cell invasion by hypoxia-inducible factor 1. *Cancer Res.* 2003;63(5):1138-1143.
37. Ju JA, et al. Hypoxia selectively enhances integrin $\alpha_5\beta_1$ receptor expression in breast cancer to promote metastasis. *Mol Cancer Res*. 2017;15(6):723-734.
38. Zhao T, et al. HIF-1α binding to AEG-1 promoter induced upregulated AEG-1 expression associated with metastasis in ovarian cancer. *Cancer Med*. 2017;6(5):1072-1081.
39. Wu H, et al. Hypoxia-mediated complement 1q binding protein regulates metastasis and chemoresistance in triple-negative breast cancer and modulates the PKC-NF-κB-VCAM-1 signaling pathway. *Front Cell Dev Biol*. 2021;9:607142.
40. Zhang H, et al. HIF-1-dependent expression of angiopoietin-like 4 and L1CAM mediates vascular metastasis of hypoxic breast cancer cells to the lungs. *Oncogene*. 2012;31(14): 1757-1770.
41. Samanta D, et al. Chemotherapy induces enrichment of $CD47^+$/$CD73^+$/$PDL1^+$ immune evasive triple-negative breast cancer cells. *Proc Natl Acad Sci U S A*. 2018;115(6): E1239-E1248.
42. Lu Y, et al. Isolation and characterization of living circulating tumor cells in patients by immunomagnetic negative enrichment coupled with flow cytometry. *Cancer.* 2015;121(17): 3036-3045.
43. Steinert G, et al. Immune escape and survival mechanisms in circulating tumor cells of colorectal cancer. *Cancer Res.* 2014;74(6):1694-1704.
44. Chiarella AM, Ryu YK, Manji GA, Rustgi AK. Extracellular ATP and adenosine in cancer pathogenesis and treatment. *Trends Cancer*. 2021;7(8):731-750.
45. Wang Y, et al. Hypoxia promotes ligand-independent EGF receptor signaling via hypoxia-inducible factor-mediated upregulation of caveolin-1. *Proc Natl Acad Sci U S A*. 2012;109(13): 4892-4897.
46. Hanna SC, et al. HIF-1α and HIF-2α independently activate SRC to promote melanoma metastases. *J Clin Invest.* 2013;123(5):2078-2093.
47. Zhang T, et al. Roles of HIF-1α in a novel optical orthotopic spontaneous metastatic bladder cancer animal model. *Urol Oncol*. 2012;30(6):928-935.

48. Krutilina RI, et al. HIF-dependent CKB expression promotes breast cancer metastasis, whereas cyclocreatine therapy impairs cellular invasion and improves chemotherapy efficacy. *Cancers (Basel)*. 2021;14(1):27.
49. Xiang L et al. Glutaminase 1 expression in colorectal cancer cells is induced by hypoxia and required for tumor growth, invasion, and metastatic colonization. *Cell Death Dis*. 2019;10(2):40.
50. Gilkes DM, et al. Hypoxia-inducible factors mediate coordinated RhoA-ROCK1 expression and signaling in breast cancer cells. *Proc Natl Acad Sci U S A*. 2014;111(3): E384-E393.
51. Martens LK, Kirschner KM, Warnecke C, Scholz H. Hypoxia-inducible factor 1 (HIF-1) is a transcriptional activator of the TrkB neurotrophin receptor gene. *J Biol Chem*. 2007;282(19): 14379-14388.
52. Sinkevicius KW, et al. Neurotrophin receptor TrkB promotes lung adenocarcinoma metastasis. *Proc Natl Acad Sci U S A*. 2014;111(28):10299-10304.
53. Goradel NH et al. Cyclooxygenase-2 in cancer: a review. *J Cell Physiol*. 2019;234(5): 5683-5699.
54. Kaidi A et al. Direct transcriptional up-regulation of cyclooxygenase 2 by hypoxia-inducible factor (HIF)-1 promotes colorectal tumor cell survival and enhances HIF-1 transcriptional activity during hypoxia. *Cancer Res*. 2006; 66(13):6683-6691.
55. Schito L, et al. Hypoxia-inducible factor 1-dependent expression of platelet-derived growth factor B promotes lymphatic metastasis of hypoxic breast cancer cells. *Proc Natl Acad Sci U S A*. 2012; 109(40):E2707-E2716.
56. Forsythe JA, et al. Activation of vascular endothelial growth factor gene transcription by hypoxia-inducible factor 1. *Mol Cell Biol*. 1996;16(9):4604-4613.
57. Erler JT, et al. Lysyl oxidase is essential for hypoxia-induced metastasis. *Nature.* 2006; 440(7088):1222-1226.
58. Wong CC, et al. Hypoxia-inducible factor 1 is a master regulator of breast cancer metastatic niche formation. *Proc Natl Acad Sci U S A*. 2011;108(39):16369-16374.
59. Schietke R, et al. The lysyl oxidases LOX and LOXL2 are necessary and sufficient to repress E-cadherin in hypoxia: insights into cellular transformation processes mediated by HIF-1. *J Biol Chem*. 2010;285(9):6658-6669.
60. Grebhardt S et al. Hypoxia and HIF-1 increase S100A8 and S100A9 expression in prostate cancer. *Int J Cancer.* 2012;131(12):2785-2794.
61. Lukanidin E, Sleeman JP. Building the niche: the role of the S100 proteins in metastatic growth. *Semin Cancer Biol*. 2012;22(3):216-225.
62. Samanta D, et al. Hypoxia-inducible factors are required for chemotherapy resistance of breast cancer stem cells. *Proc Natl Acad Sci U S A*. 2014;111(50):E5429-E5438.
63. Di Desidero T et al. Potent efficacy of metronomic topotecan and pazopanib combination therapy in preclinical models of primary or late stage metastatic triple-negative breast cancer. *Oncotarget*. 2015;6(40):42396-42410.
64. Lu H, et al. Chemotherapy triggers HIF-1-dependent glutathione synthesis and copper chelation that induces the breast cancer stem cell phenotype. *Proc Natl Acad Sci U S A*. 2015;112(33): E4600-E4609.
65. Zhang C, et al. Hypoxia induces the breast cancer stem cell phenotype by HIF-dependent and ALKBH5-mediated m^6A-demethylation of NANOG mRNA. *Proc Natl Acad Sci U S A*. 2016; 113(14):E2047-E2056.
66. Zhang C, et al. Hypoxia-inducible factors regulate pluripotency factor expression by ZNF217- and ALKBH5-mediated modulation of RNA methylation in breast cancer cells. *Oncotarget*. 2016;7(40):64527-64542.
67. Lu H et al. Reciprocal regulation of DUSP9 and DUSP16 expression by HIF-1 controls ERK and p38 MAP kinase activity and mediates chemotherapy-induced breast cancer stem cell enrichment. *Cancer Res*. 2018;78(15):4191-4202.
68. Lu H, et al. Chemotherapy-induced Ca^{2+} release stimulates breast cancer stem cell enrichment. *Cell Rep*. 2017;18(8):1946-1957.
69. Samanta D, et al. PHGDH expression is required for mitochondrial redox homeostasis, breast cancer stem cell maintenance, and lung metastasis. *Cancer Res*. 2016;76(15):4430-4442.
70. Xiang L, et al. Hypoxia-inducible factor 1 mediates TAZ expression and nuclear localization to induce the breast cancer stem cell phenotype. *Oncotarget*. 2014;5(24):12509-12527.
71. Lu H, et al. HIF-1 recruits NANOG as a coactivator for *TERT* gene transcription in hypoxic breast cancer stem cells. *Cell Rep*. 2021;36(13):109757.
72. Lu H, et al. Chemotherapy triggers HIF-1-dependent glutathione synthesis and copper chelation that induces the breast cancer stem cell phenotype. *Proc Natl Acad Sci U S A*. 2015;112(33): E4600-E4609.
73. Zhang H, et al. HIF-1 regulates CD47 expression in breast cancer cells to promote evasion of phagocytosis and maintenance of cancer stem cells. *Proc Natl Acad Sci U S A*. 2015;112(45): E6215-E6223.
74. Lan J et al. Hypoxia-inducible factor 1-dependent expression of adenosine receptor 2B promotes breast cancer stem cell enrichment. *Proc Natl Acad Sci U S A*. 2018;115(41):E9640-E9648.
75. Liu X et al. HIF-1-regulated expression of calreticulin promotes breast tumorigenesis and progression through Wnt/β-catenin pathway activation. *Proc Natl Acad Sci U S A*. 2021; 118(44):e2109144118.

76. Lu H, et al. Chemotherapy-induced S100A10 recruits KDM6A to facilitate OCT4-mediated breast cancer stemness. *J Clin Invest*. 2020;130(9):4607-4623.
77. Chen J et al. Hypoxia potentiates Notch signaling in breast cancer leading to decreased E-cadherin expression and increased cell migration and invasion. *Br J Cancer*. 2010; 102(2):351-360.
78. Cavadas MA et al. REST is a hypoxia-responsive transcriptional repressor. *Sci Rep*. 2016; 6:31355.
79. Mao XG et al. Overexpression of ZNF217 in glioblastoma contributes to the maintenance of glioma stem cells regulated by hypoxia-inducible factors. *Lab Invest*. 2011;91(7): 1068-1078.
80. Lee HY et al. HIF-1-dependent induction of Jumonji domain-containing protein (JMJD) 3 under hypoxic conditions. *Mol Cells*. 2014;37(1):43-50.
81. Wu MZ et al. Hypoxia drives breast tumor malignancy through a TET-TNFα-p38-MAPK signaling axis. *Cancer Res*. 2015;75(18):3912-3924.
82. Mariani CJ et al. TET1-mediated hydroxymethylation facilitates hypoxic gene induction in neuroblastoma. *Cell Rep*. 2014;7(5):1343-1352.

Supplemental Table 3. HIF target genes encoding mediators of immune evasion.

Gene	Protein	Mechanism of immune evasion	A/S/M*	References
BIRC2	BIRC2	Blocks NK and T cell recruitment	S	1, 2
CA9	CA9	Creates immunosuppressive acidic TME#	S/M	3-5
CCL20	CCL20	Mediates Treg recruitment		6, 7
CCL26	CCL26	Mediates recruitment of MDSCs and TAMs		8
CCL28	CCL28	Mediates Treg recruitment	A	9
CD47	CD47	Blocks phagocytosis of cancer cells	S	10-12
CD70	CD70	Induces T cell apoptosis	S	13-15
CD274	PDL1	Induces T cell exhaustion		11, 16, 17
CXCL1	CXCL1	Mediates recruitment of TAMs	A	18-20
CXCL5	CXCL5	Recruits immunosuppressive TANs^		21
CXCL12	SDF1	Recruits TAMs and MDSCs	A	22, 23
GAL3ST1	GCST	Increases platelet binding to cancer cells		24
NT5E	CD73	Generates immunosuppressive adenosine	A/S/M	11, 25-29
ENTPD1	CD39	Generates substrate for CD73	A	11, 29, 30
ENTPD2	CD39L1	Generates immunosuppressive AMP	M	29, 31
HAVCR2	TIM3	Induces T cell exhaustion		32-34
HLA-G	HLA-G	Inhibits NK and T cells		35, 36
IL10	IL10	Maintains Tregs; induces TAM polarization<br>Inhibits dendritic cells	A	37-41
IL23	IL23	Stimulates Treg proliferation<br>Increases IL10 and TGFb expression	M	42
LDHA	LDHA	Inhibits NK and T cell activity	M	43-45
MIR-25/93	mIR-25/93	Inhibits cGAS+ expression		46
NAMPT	NAMPT	Induces PDL1 expression	M	47, 48
NANOG	NANOG	Increases TGF-β** expression	S	49, 50
PDK1	PDK1	Inhibits pyruvate oxidation	M	51, 52
PGF	PGF	Promotes polarization of TAMs<br>Inhibits DC maturation	A	53
SLC2A1	GLUT1	Mediates glucose uptake	M	54, 55
SLC6A8	SLC6A8	Induces macrophage polarization	M	56, 57
SLC16A3	*MCT4*	Pumps lactate out of cancer cells	M	58
STC1	STC1	Inhibits phagocytosis and T cell activation	S	59, 60
VEGFA	VEGF-A	Recruits TAMs; inhibits DC maturation	A	61-64
VSIR	VISTA	Promotes MDSC activity		65
VTCN1	B7-H4	Inhibits T cell proliferation<br>Inhibits T cell cytokine production		66, 67

*A, angiogenesis; S, cancer stem cell specification; M, metabolism
#TME, tumor microenvironment
^TANs, tumor-associated neutrophils
+cGAS, cyclic guanosine monophosphate synthase
** TGF-β, transforming growth factor β

References

1. Carter BZ, et al. Synergistic targeting of AML stem/progenitor cells with IAP antagonist birinapant and demethylating agents. *J Natl Cancer Inst.* 2014;106(2):djt440.
2. Samanta D, et al. BIRC2 expression impairs anti-cancer immunity and immunotherapy efficacy. *Cell Rep.* 2020;32(8):108073.
3. Giatromanolaki A, et al. Carbonic anhydrase 9 (CA9) expression in non-small-cell lung cancer: correlation with regulatory FOXP3+ T-cell tumor stroma infiltration. *Br J Cancer*. 2020;122(8): 1205-1210.
4. Ledaki I, et al. Carbonic anhydrase IX induction defines a heterogeneous cancer cell response to hypoxia and mediates stem cell-like properties and sensitivity to HDAC inhibition. *Oncotarget.* 2015;6(23):19413-19427.

5. Pastorekova S, Gillies RJ. The role of carbonic anhydrase IX in cancer: links to hypoxia, acidosis, and beyond. *Cancer Metastasis Rev*. 2019;38(1-2):65-77.
6. Wang D, et al. Colorectal cancer cell-derived CCL20 recruits regulatory T cells to promote chemoresistance via FOXO1/CEBPB/NF−κB signaling. *J Immunother Cancer*. 2019;7:215.
7. Ye LY, et al. Hypoxia-induced epithelial-to-mesenchymal transition in hepatocellular carcinoma induces an immunosuppressive tumor microenvironment to promote metastasis. *Cancer Res.* 2016; 76(4):818-830.
8. Chiu DK, et al. Hypoxia induces myeloid-derived suppressor cell recruitment to hepatocellular carcinoma through chemokine (C-C motif) ligand 26. *Hepatology*. 2016;64:797-813.
9. Facciabene A, et al. Tumor hypoxia promotes tolerance and angiogenesis via CCL28 and Treg cells*.* *Nature*. 2011;475:226-230.
10. Jaiswal S, et al. Macrophages as mediators of tumor immunosurveillance. *Trends Immunol*. 2010;31:212-219.
11. Samanta D, et al. Chemotherapy induces enrichment of $CD47^+/CD73^+/PDL1^+$ immune evasive triple-negative breast cancer cells. *Proc Natl Acad Sci USA* 2018;115(6):E1239-E1248.
12. Zhang H, et al. HIF-1 regulates CD47 expression in breast cancer cells to promote evasion of phagocytosis and maintenance of cancer stem cells. *Proc Natl Acad Sci USA*. 2015;112(45): E6215-E6223.
13. Diegmann J, et al. Immune escape for renal cell carcinoma: CD70 mediates apoptosis in lymphocytes. *Neoplasia*. 2006;8(11):933-938.
14. Jacobs J, et al. CD70: an emerging target in cancer immunotherapy. *Pharmacol Ther.* 2015;155: 1-10.
15. Kitajima S, et al. Hypoxia-inducible factor-2a upregulates CD70 under hypoxia and enhances anchorage-independent growth and aggressiveness in cancer cells. *Oncotarget*. 2018;9(27): 19123-19135.
16. Barsoum IB, et al. A mechanism of hypoxia-mediated escape from adaptive immunity in cancer cells. *Cancer Res*. 2014;74(3):665-674.
17. Noman MZ, et al. PD-L1 is a novel direct target of HIF-1a and its blockade under hypoxia enhanced MDSC-mediated T cell activation. *J Exp Med*. 2014;211(5):781-790.
18. Korbecki J, et al. The effect of hypoxia on the expression of CXC chemokines and CXC chemokine receptors-a review of literature. *Int J Mol Sci.* 2021;22(2):843.
19. Miyake M, et al. CXCL1-mediated interaction of cancer cells with tumor-associated macrophages and cancer-associated fibroblasts promotes tumor progression in human bladder cancer. *Neoplasia*. 2016;18(10):636-646.
20. Scortegagna M, et al. HIF-1α regulates epithelial inflammation by cell autonomous NF-kB activation and paracrine stromal remodeling. *Blood*. 2008;111(7):3343-3354.
21. Zhou SL, et al. Tumor-associated neutrophils recruit macrophages and T-regulatory cells to promote progression of hepatocellular carcinoma and resistance to sorafenib. *Gastroenterology* 2016;150:1646-1658.
22. Chen Y, et al. Differential effects of sorafenib on liver versus tumor fibrosis mediated by stromal-derived factor 1α/C-X-C receptor type 4 axis and myeloid differentiation antigen-positive myeloid cell infiltration in mice. *Hepatology* 2014;59:1435-1447.
23. Du R, et al. HIF-1α induces the recruitment of bone marrow-derived vascular modulatory cells to regulate tumor angiogenesis and invasion. *Cancer Cell*. 2008;13:206-220.
24. Robinson CM, et al. A hypoxia-inducible HIF1-GAL3ST1-sulfatide axis enhances ccRCC immune evasion via increased tumor cell-platelet binding. *Mol Cancer Res*. 2019;17:2306-2317.
25. Antonioli L, et al. Adenosine signaling in the tumor microenvironment. *Adv Exp Med Biol*. 2021; 1270:145-167.
26. Sitkovsky MV, et al. Hostile, hypoxia-A2-adenosinergic tumor biology as the next barrier to overcome for tumor immunologists. *Cancer Immunol Res*. 2014;2(7);598-605.
27. Soleimani A, et al. CD73, a key ectonucleotidase in the development of breast cancer: recent advances and perspectives. *J Cell Physiol.* 2019;234(9):14622-14632x
28. Synnestvedt K, et al. Ecto-5'-nucleotidase (CD73) regulation by hypoxia-inducible factor-1 mediates permeability changes in intestinal epithelia. *J Clin Invest*. 2002;110(7):993-1002.
29. Yuen VW, Wong CC. Hypoxia-inducible factors and innate immunity in liver cancer. *J Clin Invest*. 2020;130(10):5052-5062.
30. Hatfield SM, Sitkovsky M. A2A adenosine receptor antagonists to weaken the hypoxia-HIF-1α driven immunosuppression and improve immunotherapies of cancer. *Curr Opin Pharmacol*. 2016;29:90-96.
31. Chiu DK, et al. Hypoxia-inducible factor HIF-1 promotes myeloid-derived suppressor cells accumulation through ENTPD2/CD39L1 in hepatocellular carcinoma. *Nat Commun*. 2017;8: 517.
32. Hakemi MG, et al. The role of TIM-3 in hepatocellular carcinoma: a promising target for immunotherapy? *Front Oncol*. 2020;10:601661.
33. Koh HS, et al. The HIF-1/glial TIM-3 axis controls inflammation-associated brain damage under hypoxia. *Nat Commun*. 2015;6:6340.

34. Leone P, et al. The evolving role of immune checkpoint inhibitors in hepatocellular carcinoma treatment. *Vaccines*. 2021;9(5):532.
35. Martin-Villa JM, et al. HLA-G: too much or too little? Role in cancer and autoimmune disease. *Front Immunol*. 2022;13:796054.
36. Yaghi L, et al. Hypoxia inducible factor-1 mediates the expression of the immune checkpoint HLA-G in glioma cells through hypoxia response element located in exon 2. *Oncotarget*. 2016; 7(39):63690–63707.
37. Bogdan C, et al. Macrophage deactivation by interleukin 10. *J Exp Med*. 1991;174:1549.
38. Dace DS, et al. Interleukin-10 promotes pathological angiogenesis by regulating macrophage response to hypoxia during development. *PLoS One.* 2008;3:e3381.
39. Murai M, et al. Interleukin 10 acts on regulatory T cells to maintain expression of the transcription factor Foxp3 and suppressive function in mice with colitis. *Nat Immunol*. 2009; 10:1178-1184.
40. Storti P, et al. Hypoxia-inducible factor (HIF)-1α suppression in myeloma cells blocks tumoral growth in vivo inhibiting angiogenesis and bone destruction. Leukemia. 2013;27(8):1697-1706.
41. Tran CW, et al. Hypoxia-inducible factor 1α limits dendritic cell stimulation of CD8 T cell immunity. *PLoS One*. 15(12):e0244366.
42. Fu Q, et al. Tumor-associated macrophage-derived interleukin-23 interlinks kidney cancer glutamine addiction with immune evasion. *Eur Urol*. 2019;75(5):752-763.
43. Brand A, et al. LDHA-associated lactic acid production blunts tumor immunosurveillance by T and NK cells. *Cell Metab*. 24(2016):657-671.
44. Fischer K, et al. Inhibitory effect of tumor cell-derived lactic acid on human T cells. *Blood*. 2007;109:3812-3819.
45. Semenza GL, et al. Hypoxia response elements in the aldolase A, enolase 1, and lactate dehydrogenase A gene promoters contain essential binding sites for hypoxia-inducible factor 1. *J Biol Chem*. 1996;271(51):32529-32537.
46. Wu MZ, et al. miR-25/93 mediates hypoxia-induced immunosuppression by repressing cGAS. *Nat Cell Biol*. 2017;19:1286-1296.
47. Lv H, et al. NAD^+ metabolism maintains inducible PD-L1 expression to drive tumor immune evasion. *Cell Metab*. 2021;33:1-8.
48. Segawa K, et al. Visfatin in adipocytes is upregulated by hypoxia through HIF-1α-dependent mechanism. *Biochem Biophys Res Commun*. 2006;349(3):875-882.
49. Hasmim M, et al. Hypoxia-dependent inhibition of tumor cell susceptibility to CTL-mediated lysis involves NANOG induction in target cells. *J Immunol*. 2011;187:4031-4039.
50. Xiang L, Semenza GL. Hypoxia-inducible factors promote breast cancer stem cell specification and maintenance in response to hypoxia or cytotoxic chemotherapy. *Adv Cancer Res*. 2019; 141:175-212.
51. Kim JW, et al. HIF-1-mediated expression of pyruvate dehydrogenase kinase: a metabolic switch required for cellular adaptation to hypoxia. *Cell Metab*. 2006;3:177-185.
52. Papandreou I, et al. HIF-1 mediates adaptation to hypoxia by actively downregulating mitochondrial oxygen consumption. *Cell Metab*. 2006;3:187-197.
53. Voron T, et al. Control of the immune response by pro-angiogenic factors. *Front Oncol*. 2014;4: 70.
54. Chang CH, et al. Metabolic competition in the tumor microenvironment is a driver of cancer progression. *Cell* 2015;162:1229-1241.
55. Ottensmeier CH, et al. Upregulated glucose metabolism correlates inversely with CD8+ T cell infiltration and survival in squamous cell carcinoma. *Cancer Res.* 2016;76(14):4136-4148.
56. Ji L, et al. Slc6a8-mediated creatine uptake and accumulation reprogram macrophage polarization via regulating cytokine responses. *Immunity*. 2019;51:272-284.
57. Li Y, et al. Stanniocalcin-1 augments stem-like traits of glioblastoma cells through binding and activating NOTCH1. *Cancer Lett*. 2018:416:66-74.
58. Halestrap AP, Wilson MC. The monocarboxylate transporter family – role and regulation. *IUBMB Life*. 2012;64(2):109-119.
59. Lin H, et al. Stanniocalcin 1 is a phagocytosis checkpoint driving tumor immune resistance. *Cancer Cell.* 2021;39:480-493.
60. Manalo DJ, et al. Transcriptional regulation of vascular endothelial cell responses to hypoxia by HIF-1. *Blood*. 2005;105(2):659-669.
61. Dineen SP, et al. Vascular endothelial growth factor receptor 2 mediates macrophage infiltration into orthotopic pancreatic tumors in mice. *Cancer Res*. 2008;68:4340-4346.
62. Forsythe JA, et al. Activation of vascular endothelial growth factor gene transcription by hypoxia-inducible factor 1. *Mol Cell Biol*. 1996;16(9):4604-4613.
63. Gabrilovich D, et al. Vascular endothelial growth factor inhibits the development of dendritic cells and dramatically affects the differentiation of multiple hematopoietic lineages. *Blood.* 1998;92: 4150-4166.

64. Yang J, Yan IJ, Liu B. Targeting VEGF/VEGFR to modulate anti-tumor immunity. *Front Immunol*. 2018;9:978.
65. Dang J, et al. Hypoxia-induced VISTA promotes the suppressive function of myeloid-derived suppressor cells in the tumor microenvironment. *Cancer Immunol Res.* 2019;7(7):1079-1090.
66. Jeon YK, et al. Cancer cell-associated cytoplasmic B7-H4 is induced by hypoxia through hypoxia-inducible factor-1a and promotes cancer cell proliferation. *Biochem Biophys Res Commun*. 2015;459:277-283.
67. MacGregor HL, Ohashi PS. Molecular pathways: evaluating the potential for B7-H4 as an immunoregulatory target. *Clin Cancer Res*. 2017;23(12):2934-2941.

Supplemental Table 4. Clinical trials of belzutifan registered at ClinicalTrials.gov.

NCT Identifier	Tumor type(s)[&]	Ph[#]	n[^]	Drugs[*]
02974738	Advanced solid tumors			Belzutifan
03401788	VHL-associated RCC	2	50	Belzutifan
03634540	ccRCC	2	118	Belzutifan + Cabozantinib
04195750	Advanced RCC	3	736	Belzutifan vs Everolimus
04489771	Advanced RCC	2	150	Belzutifan
04736706	Advanced ccRCC	3	1431	Belz + Pemb + Lenv vs Pemb + Lenv
04846920	Advanced ccRCC	1	52	Belzutifan
04924075	Pheochromocytoma<br>Paraganglioma<br>Pancreatic neuroendocrine	1	140	Belzutifan
04976634	Solid tumors	2	400	Belz + Pemb + Lenv
05030506	Advanced RCC	1	45	Belz + Pemb + Lenv
05239728	ccRCC s/p nephrectomy	3	1600	Belz + Pemb vs Pemb

[&]RCC, renal cell carcinoma; ccRCC, clear cell RCC
[#]Ph, phase 1, 2, or 3 trial
[^]n, number of subjects
[*]drug abbreviations: belzutifan (Belz), cabozantinib (Cabo), lenvatinib (Lenv), and pembrolizumab (Pemb)
